# Alternative Models for Anticancer Drug Discovery From Natural Products Using Binary Tumor‐Microenvironment‐on‐a‐Chip

**DOI:** 10.1002/advs.202507944

**Published:** 2025-07-28

**Authors:** Youngwon Kim, Si Hyeon Chae, Dahae Lee, Bum Soo Lee, Jiseok Lim, Hyo‐Il Jung, Ki Hyun Kim, Bongseop Kwak

**Affiliations:** ^1^ School of Mechanical Engineering Yonsei University 50 Yonsei‐ro, Seodaemun‐gu Seoul 13722 Republic of Korea; ^2^ College of Medicine Dongguk University 32 Dongguk‐ro, Ilsandong‐gu Goyangsi Gyeonggi‐do 10326 Republic of Korea; ^3^ School of Pharmacy Sungkyunkwan University Suwon 16419 Republic of Korea; ^4^ College of Korean Medicine Gachon University Seongnam 13120 Republic of Korea; ^5^ School of Mechanical Engineering Yeungnam University 280 Daehak‐ro Gyeongsan‐si Gyeongsangbuk‐do 38541 Republic of Korea; ^6^ MediSphere Inc., 280, Daehak‐ro Gyeongsan‐si Gyeongsangbuk‐do 38541 Republic of Korea; ^7^ The DABOM Inc. 50 Yonsei‐ro Seodaemun‐gu Seoul 03722 Republic of Korea

**Keywords:** alternative model, anticancer natural products, binary tumor‐microenvironment‐on‐a‐chip (binary T‐MOC), new approach methodologies (NAMs), poisonous mushrooms

## Abstract

The efficacy evaluation of anticancer drugs derived from natural products has traditionally relied on animal models, highlighting the need for more efficient preclinical assessment platforms. In this study, a binary tumor‐microenvironment‐on‐a‐chip (T‐MOC) system is introduced to assess the therapeutic potential of illudin S and roridin E, two cytotoxic compounds derived from *Omphalotus japonicus* and *Podostroma cornu‐damae*, respectively. The binary T‐MOC model integrates independently developed vascular and invasive ductal carcinoma compartments, effectively mimicking in vivo drug delivery barriers and physiological dynamics. Using this model, illudin S demonstrates strong anticancer effects but exhibits high toxicity, particularly in the lung and liver, indicating a narrow therapeutic window. Roridin E demonstrates potent activity at low concentrations but exhibits high toxicity, especially in the liver and skin. Additionally, morphological analysis is performed to predict drug delivery and distribution characteristics, revealing anisotropic remission and the influence of microenvironmental factors on drug response. This study underscores the potential of the binary T‐MOC system as an alternative platform for anticancer drug evaluation, enabling efficient preclinical validation while reducing reliance on animal models.

## Graphical abstract

This study presents a binary tumor‐microenvironment‐on‐a‐chip (T‐MOC) system incorporating multicellular tumor spheroids (MCTs) as an alternative preclinical platform to evaluate the efficacy of anticancer natural products. The T‐MOC model reproduces in vivo drug delivery barriers and physiological conditions, enabling morphological analysis to predict drug delivery and distribution characteristics, thereby highlighting its potential to reduce reliance on animal testing.

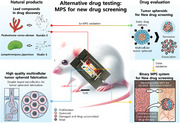

## 1 Introduction

The discovery and development of lead compounds for clinical use require a thorough evaluation of efficacy, pharmacokinetics, pharmacodynamics, and potential toxicity in specific organs.^[^ ^1^ ^]^ Traditionally, such assessments have relied on animal models,^[^ ^2^ ^]^ which, despite their physiological relevance, present several challenges, including interspecies differences,^[^ ^3^ ^]^ high costs, ethical concerns, and limited throughput.^[^ ^4^ ^]^ To address these challenges, the Food and Drug Administration Modernization Act 2.0 has advocated for the adoption of new approach methodologies (NAMs), such as organoids and microphysiological systems (MPS),^[^ ^5^ ^]^ which aim to overcome the limitations of traditional models while providing a more biomimetic environment.^[^ ^6^ ^]^ NAMs enable more accurate predictions of drug efficacy and toxicity, reduce unnecessary animal testing, and facilitate drug evaluation in alignment with the 3Rs principle (replacement, reduction, and refinement).^[^ ^7^ ^]^ In oncology research, commonly used in vitro NAMs include multicellular tumor spheroids (MCTs) and MPS‐based tumor‐microenvironment‐on‐a‐chip (T‐MOC) models, which are employed to assess the efficacy of anticancer lead compounds, and optimize dose concentrations. MCTs replicate key tumor characteristics, including high cell density, hypoxia, and cell–cell interactions, which collectively contribute to drug resistance.^[^ ^8^ ^]^ T‐MOC systems further integrate MCTs with their microenvironment, incorporating the extracellular matrix (ECM), vasculature, and physiological dynamics to closely mimic in vivo tumor conditions.^[^ ^9^ ^]^ These features enable a more precise replication of the in vivo tumor microenvironment, improving drug efficacy predictions.^[^ ^10^ ^]^

Numerous natural product‐based anticancer drugs, such as paclitaxel, doxorubicin (DOX), camptothecin, vinblastine, and vincristine, have been successfully developed and are widely used in clinical practice.^[^ ^11^ ^]^ However, the discovery of new anticancer agents remains essential due to emerging drug resistance, severe side effects, and the need for improved therapeutic efficacy. Despite the promising cytotoxic potential of many natural compounds, their clinical development requires a well‐defined therapeutic index and a detailed understanding of their mechanisms of action to ensure both efficacy and safety. Without clear elucidation of their mechanisms, including the identification of molecular targets and signaling pathways, the therapeutic applicability of these compounds remains uncertain.^[^ ^12^ ^]^ Additionally, thorough validation of toxicity profiles is crucial, as off‐target effects can significantly limit clinical use, regardless of a compound's anticancer potential. Recent research on plant‐derived anticancer compounds emphasizes their selectivity for cancer cells while preserving normal tissues, reinforcing their viability as therapeutic candidates.^[^ ^13^ ^]^ However, some compounds, like dihydromyricetin, show therapeutic promise against liver cancer by regulating apoptosis, autophagy, and redox balance, yet require further translational research.^[^ ^14^ ^]^ Additionally, natural products have traditionally been associated with challenges such as low production yields and difficulties in maintaining consistent quality. These limitations hinder their advancement into preclinical stages, where large quantities of standardized compounds are required. Thus, while anticancer natural products offer promising therapeutic avenues, their clinical success depends on rigorous validation of efficacy, comprehensive toxicity assessment, and detailed mechanistic studies. To address limitations, we employed a binary T‐MOC to evaluate the anticancer effects of natural products illudin S and roridin E, derived from the poisonous mushrooms *Omphalotus japonicus* and *Podostroma cornu‐damae*, respectively. T‐MOC platforms commonly employ architectures such as self‐assembly, sandwich, and multichannel systems. However, challenges remain in co‐culturing, the time‐consuming process of forming the microenvironment and maturation, low fabrication yield, and the complexity of controlling physiological dynamics within the system. The binary T‐MOC model addresses traditional limitations in productivity and co‐culture conditions by enabling independent part development, thereby streamlining the experimental process. Notably, efficacy evaluation in this system requires only 0.1% to 0.2% of the drug quantity typically used in animal experiments for full‐scale testing, making it particularly suitable for assessing the therapeutic potential of low‐yield natural compounds. This system facilitates the retrieval of post‐treatment samples, enabling efficient downstream analysis and supporting the advancement of alternative models to animal testing. It precisely evaluates drug efficacy by simulating the in vivo environment and assessing drug responsiveness at optimal concentrations. In addition, drug mechanisms, distribution, and accumulation, combined with physiological dynamics, can significantly influence the morphological characteristics of MCTs, providing insights into drug response patterns.^[^ ^15^ ^]^ Building on these observations, we propose geometry‐based parameters to enhance drug evaluation and conduct a comparative analysis of efficacy across different in vitro models. Enhancing the physiological relevance of in vitro models and diversifying evaluation metrics may reduce reliance on animal studies and facilitate more efficient validation of new drug candidates within the therapeutic ranges. In this context, the binary T‐MOC system provides a promising platform for evaluating natural product‐based anticancer agents in a physiologically dynamic environment.

## 2 Result and Discussion

### 2.1 Validation of Binary T‐MOC System and Consistency With Animal Models

**Figure 1:**
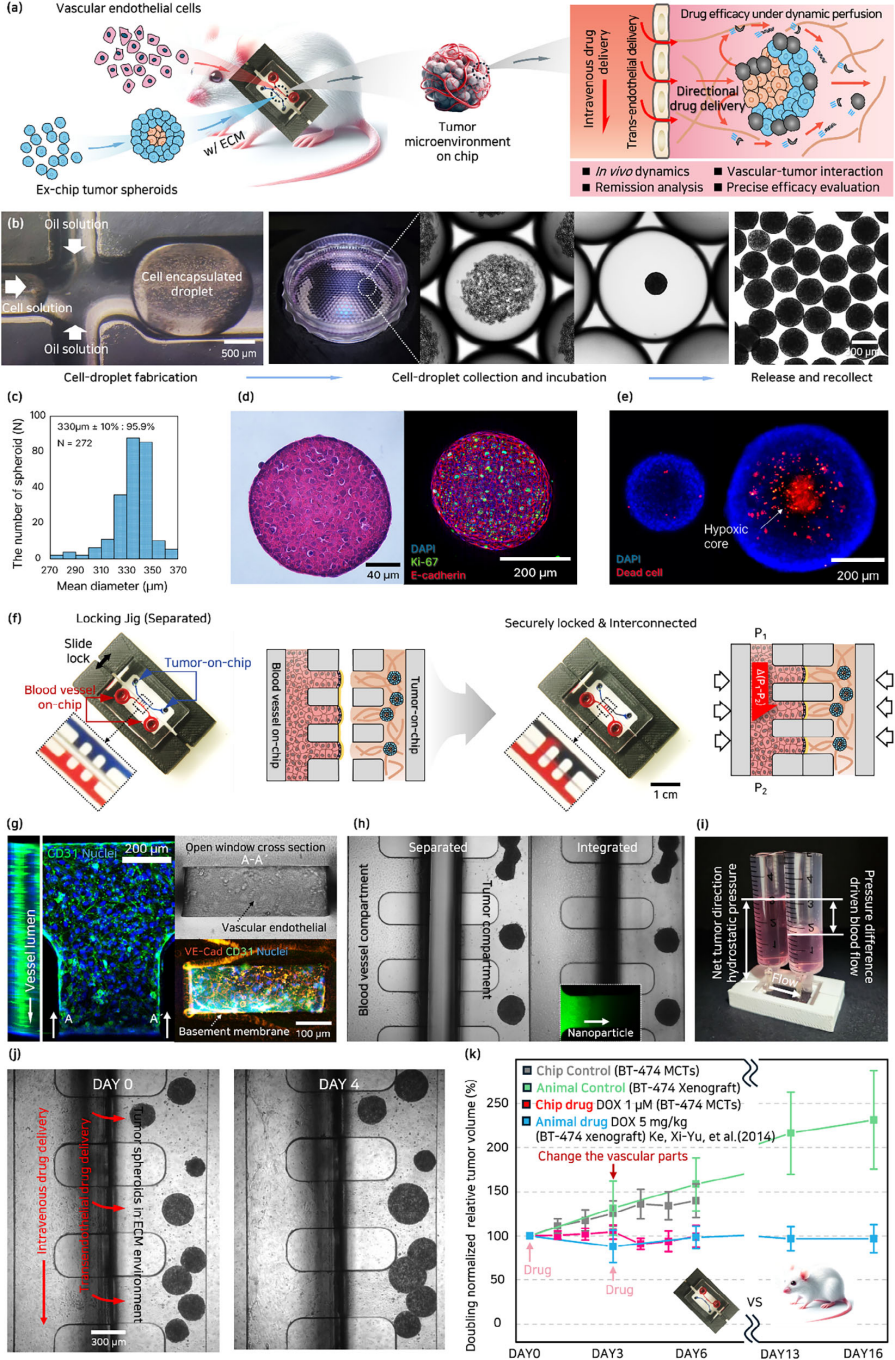
Overview of the binary tumor microenvironment‐on‐a‐chip. A) Schematic illustration of the binary T‐MOC designed for anticancer drug efficacy evaluation. The system mimics the tumor microenvironment by incorporating a vascular compartment and MCTs embedded within an ECM matrix. It provides in vivo–like dynamics and vascular–tumor interactions on the platform, supporting both intravenous and trans‐endothelial drug delivery. This enables remission analysis and precise evaluation of anticancer efficacy. B) Mass production of MCTs using a droplet‐based microfluidic system. Tumor cells suspended in medium are sheared into droplets by the oil phase. After 72 h of culture, fully developed MCTs are formed within the droplets. C) Collected MCTs underwent size distribution analysis, supporting precise validation of drug efficacy in standard breast cancer chemotherapy regimens. D) To assess the maturity and structural integrity of the MCTs, H&E staining and immunofluorescence analysis were performed. The expression of tight junction and proliferation markers, including Ki‐67 and E‐cadherin, was confirmed. Immunofluorescence staining visualized these markers: Ki‐67 (green), E‐cadherin (red), and nuclei (blue). E) Live/Dead fluorescence staining was performed to assess hypoxia in MCTs of varying sizes. Nuclei were labeled with Hoechst 33342 (blue), and dead cells were identified using propidium iodide (red). F) Optical image of the fabricated and assembled binary T‐MOC. Red and blue dyes were used to visualize the vascular and tumor compartments, respectively. The two PDMS‐based components were joined using a slide‐locking jig that applied mechanical compression, and the elastomeric and adhesive properties of PDMS ensured a leak‐free interconnection. A schematic illustration depicts the fabrication process, in which the monolithic device was divided into two compartments: the vascular side, where endothelium was developed on a freestanding scaffold, and the tumor side, consisting of ECM‐laden BT‐474 MCTs. The two mature compartments were then reassembled using the jig. G) Validation of endothelial tight junction integrity and vessel lumen structure using confocal microscopy, along with cross‐sectional views of the open vascular channel obtained through conventional fluorescence microscopy. H) Individually developed vascular and tumor compartments were integrated to form a tumor microenvironment model. Intercompartmental exchange was confirmed by the diffusion of 40 kDa dextran nanoparticles. Schematic of the fluidic setup used for perfusion‐based culture in the binary T‐MOC system. I) Syringes connected to inlet‐side reservoirs delivered culture medium at a constant flow rate using a syringe pump. The microchannels were coated with polydopamine and fibronectin to enhance surface hydrophilicity and reduce flow resistance. Hydrostatic pressure differences between the ECM and vascular compartments were adjusted to mimic physiological flow conditions. J) Structural overview of the binary T‐MOC, composed of a vascular channel and ECM‐laden MCTs compartment. Anticancer drugs are delivered across the endothelium via diffusion, recapitulating in vivo blood flow and pressure dynamics. K) Validation of the binary T‐MOC system by comparison with an IDC xenograft mouse model. DOX‐induced vascular damage in the T‐MOC led to compromised perfusion, necessitating replacement of the vascular compartment with a newly developed healthy endothelium on day 3 for second‐line treatment. Statistical analysis revealed a significant difference (*p* < 0.05) between the chip and animal control groups, whereas no significant difference (*p* > 0.05) was observed among the drug‐treated groups.

In vitro models exhibit distinct productivity and physiological characteristics, offering valuable insights into drug responses. (Figure S1, Supporting Information) Developing such alternative models is essential for the effective evaluation of drug efficacy. The binary T‐MOC model consists of a tumor compartment —comprising MCTs and the surrounding ECM—and a vascular compartment formed by vascular endothelial cells. These two components are aligned face‐to‐face using precision jigs, enabling the system to replicate the in vivo drug delivery barrier. Within the tumor compartment, the MCTs developed in this study serve as physiologically relevant models, closely mimicking the 3D architecture and gene expression profiles of solid tumors.^[^ ^15^ ^]^ Anticancer drugs exert therapeutic efficacy by inhibiting disease progression, thereby inducing remission, which refers to a reduction in disease symptoms. Drug‐induced remission may further lead to tumor disruption, characterized by the breakdown or disintegration of tumor tissue. In the binary T‐MOC model, the microchannel architecture and spatial positioning of MCTs within the ECM influence the fluid streamlines and particle trajectories. Consequently, alterations in drug diffusion, flow directionality, and microenvironmental gradients emerge, giving rise to anisotropic patterns of tumor remission. Furthermore, integration with the vascular compartment enables the simulation of physiological dynamics, allowing for the evaluation of drug efficacy, delivery, accumulation, and tumor remission under convection forces such as blood and interstitial flow. This dynamic interplay between the vascular system and the MCTs also facilitates the observation of distinct morphological transitions in response to drug exposure (**Figure** **1**a).

In drug screening and efficacy testing using MCTs, the effectiveness of the drug varies depending on the size of the MCTs and the degree of tumor development (Figure S2, Supporting Information). Therefore, mass fabrication of uniformly sized and fully developed MCTs is important in accurately evaluating drugs. However, traditional methods (U‐shape microstructure, hanging drop, Micropillar arrays, etc.) have limitations in terms of low production yield and quality.^[^ ^16^ ^]^ In this study, to overcome this challenge, MCTs were fabricated using a droplet‐based microfluidics system with distinct phases of solutions.^[^ ^8b^ ^]^ Breast tumor cells suspended in a culture media solution were encapsulated in droplets by an oil‐phase solution and cultured for three days for the full development of the MCTs. Large MCTs (>500 µm in diameter) can replicate several characteristics of human solid tumors, including the development of hypoxic cores that lead to the upregulation of hypoxia‐inducible factors.^[^ ^17^ ^]^ This hypoxic environment induces drug resistance mechanisms, including increased expression of efflux transporters and altered drug metabolism pathways that are not typically observed in 2D cultures. Additionally, hypoxia‐driven glycolytic shifts result in lactate accumulation and acidification of the spheroid core, both of which are known to impair drug uptake.^[^ ^18^ ^]^

To minimize hypoxia‐induced bias in drug efficacy evaluation, spheroid sizes were selected based on tumor growth curves to maintain physiological relevance while avoiding the onset of severe hypoxia. This approach ensures a more reliable assessment of anticancer drug responses under conditions that closely mimic the in vivo tumor microenvironment.^[^ ^8b^ ^]^ The size distribution of mass‐fabricated MCTs showed that 95.9% of the 272 MCTs had an optimized diameter of 330 µm ± 10%, indicating a highly uniform population suitable for drug evaluation. (Figure 1b,c) The quality and precise size of MCTs, along with the presence or absence of a necrotic core, were assessed to confirm structural integrity and experimental suitability (Figure 1d,e).

Conventionally, organ‐on‐a‐chip face challenges in co‐culturing cells or organs due to varying requirements for essential parts and supplements in the media, which affect functional activation and growth. Additionally, in the formation of blood vessels, the collagen structure shrinks, leading to the collapse of the ECM layer (Video S1, Supporting Information). Moreover, retrieving reaction‐completed specimens from the system for downstream analysis is challenging. Our binary system can overcome these challenges and further fully develop each organ individually, saving time consuming. The binary T‐MOC was cut into monolithic organ‐on‐a‐chip using a conventional knife cutter to separate tumor‐on‐chip and blood vessel‐on‐chip; each part can be easily interconnected using a simple slide‐locking jig that applies mechanical compression. In blood vessel parts, a basement membrane ECM solution was spread on the open side of the chip to form a free‐standing supporting structure and develop the vascular endothelium. In tumor parts, to construct the IDC MCTs laden ECM environment, IDC MCTs were suspended in a pre‐mixed collagen gel solution and injected into the tumor channel. The completely developed vascular and tumor parts were then reconnected using a jig, as shown in Figure 1f. Vascular formation was confirmed through the expression of endothelial‐specific markers such as CD31, indicating the successful development of functional vasculature. Furthermore, observation through the open vascular channel revealed the expression of endothelial tight‐junction proteins CD31 and VE‐cadherin in the absence of basement membrane rupture, further supporting the formation of a continuous vascular structure. (Figure 1g) Further details on vascular verification and additional information are described in a previous study.^[^ ^19^ ^]^ Two independently formed compartments were physically integrated to construct a single T‐MOC. The diffusion of 40 kDa dextran nanoparticles confirmed that the two channels were connected without leakage. (Figure 1h)

Traditionally, both experimental and theoretical studies have demonstrated that tumors often exhibit elevated interstitial fluid pressure (IFP), which is known to serve as a significant physical barrier impeding the transport of nutrients and small molecules within the tumor.^[^ ^20^ ^]^ However, contrary to the anticipated IFP plateau within tumors, ongoing research has shown that interstitial fluid flow (IFF) persists, facilitating substantial convection transport.^[^ ^21^ ^]^ These findings highlight the significant impact of IFF on drug delivery within tumors, emphasizing the need for further investigation into the clinical and prognostic significance of changes in fluid flow and their relevance to cancer progression and therapy response. Nevertheless, the intricate interplay of biophysical factors, including vascular structure, permeability, fluid dynamics, and drug transport mediated by diffusion and convection, remains poorly understood.^[^ ^22^ ^]^ Therefore, by employing a T‐MOC, we were able to observe and propose the role of IFF in drug delivery and remission. The culture medium was perfused to the vascular channel to mimic physiological dynamics within the body, with a capillary velocity of 0.8 mm s^−1^ (in vivo: 0.5–1.5 mm s^−1^) ^[^ ^23^ ^]^ and net tumor hydrostatic pressure of 1.47–2.2 mmHg (in vivo: −10–10 mmHg).^[^ ^24^ ^]^ Syringes were connected to the reservoirs of each channel, and a syringe pump was employed to deliver culture medium at a constant flow rate into the inlet‐side reservoirs of the chip. This configuration enabled the simulation of physiological flow within the vascular compartment and the generation of net tumor hydrostatic pressure. To minimize flow resistance and ensure stable perfusion, the microchannels were pre‐coated with polydopamine and fibronectin to enhance surface hydrophilicity. By modulating the hydrostatic pressure gradient between the organoid‐laden ECM and the vascular channel, the system can be tuned to recreate organ‐specific microenvironments. This approach extends the platform's applicability beyond MCTs to a broader range of tissue models. The underlying concept is based on replicating physiological dynamics through controlled hydrostatic pressure differentials. (Figure 1i) In the constructed binary T‐MOC system, culture medium supplied through the vascular channel enabled the cultivation of ECM‐laden MCTs, resulting in an observable increase in their size. (Figure 1j) The consistency of the binary T‐MOC system with the invasive ductal carcinoma (IDC) xenograft mouse model was evaluated by comparing tumor remission.^[^ ^25^ ^]^ The clinical plasma concentration of DOX for breast cancer chemotherapy ranges from 12.54 to 620.01 ng mL^−1^ (0.021–1.037 µm).^[^ ^26^ ^]^ The intravenous drug concentration in binary T‐MOC vascular channel matched the clinical concentration 1 µm, with a drug administration cycle of a 24 h intravenous injection followed by a 48 h observation period, synchronized with the xenograft model target chemotherapy regimen. To ensure the reliability of the binary T‐MOC model, we reused data from a previously published xenograft study using the BT‐474 cell line.^[^ ^25^ ^]^ In the study conducted by X.‐Y. Ke et al., BT‐474 cells were injected into the right flank of female BALB/c nude mice to establish a xenograft model for evaluating anticancer efficacy. The control group received 0.9% saline, while the DOX‐treated group administered DOX at a dose of 5 mg/kg. The drug was delivered intravenously on days 0, 3, 6, and 10. Relative tumor volume (RTV) measurements were obtained and compared with the corresponding results from the binary T‐MOC model to assess consistency between the two systems. DOX caused vascular damage, leading to changes in the newly developed vascular compartment before drug treatment. Since MCTs and xenograft models differ significantly in tumor growth rates and microenvironments, directly comparing their tumor volumes can lead to misinterpretation. To ensure a biologically relevant and fair evaluation, the data were normalized based on each model's volume doubling time, allowing drug responses to be compared at equivalent growth stages. Accordingly, normalization based on volume doubling time was applied to enable a valid comparison between the binary T MOC and xenograft models. In the control group, the increase in relative tumor volume (RTV) up to day 3 showed no statistically significant difference between the two models. However, a significant difference was observed on day 6, likely due to the spatial constraints within the microchannel, which altered the tumor growth trajectory as expansion continued. In contrast, tumor volume increases were suppressed in both the xenograft and binary T‐MOC models following treatment, with no statistically significant difference observed between the two models. This trend suggests a transition to a stable disease state compared to the control group. The results demonstrated a consistent tumor volume reduction trend across models, confirming the reliability of remission data (Figure 1k).

### 2.2 Evaluation of the Cytotoxicity of Natural Products Derived From the Poisonous Mushrooms *O. japonicus* and *P. cornu‐damae*

The ingestion of poisonous mushrooms can lead to a range of toxic effects, from mild gastrointestinal discomfort to severe organ failure and even fatality.^[^ ^32^ ^]^ Previous research has identified various classes of toxic compounds in these mushrooms, such as cyclic peptides, triterpenes, alkaloids, and other secondary metabolites, shedding light on their biological activities and toxicity.^[^ ^32^ ^]^ Despite their toxicity, poisonous mushrooms also exhibit intriguing bioactivities, including potential antimicrobial, anticancer, and immunomodulatory properties, which have attracted the interest of natural product chemists^[^ ^27^ ^]^ as platforms for the discovery of novel therapeutic agents. The toxic compounds derived from poisonous mushrooms have the potential to be repurposed as therapeutic agents, particularly in the context of anticancer treatments.^[^ ^27^, ^28^ ^]^ However, harnessing these toxic compounds requires careful evaluation and management of their inherent toxicity.

In line with ongoing research interest aiming to uncover anticancer natural products,^[^ ^29^ ^]^ we have turned our focus to investigating potential anticancer compounds derived from poisonous mushrooms, specifically *O. japonicus* and *P. cornu‐damae*. *O. japonicus* (Kawam.) Kirchm. & O. K. Mill is a poisonous mushroom found in Japan and Eastern Asia, belonging to the family Marasmiaceae. Known for its distinctive orange‐to‐brown fruiting bodies and bioluminescent properties, *O. japonicus* is visible in the dark (Figure S3, Supporting Information).^[^ ^30^ ^]^ Consumption of this mushroom can cause symptoms such as nausea and vomiting due to its toxic compounds.^[^ ^31^ ^]^ Sesquiterpenoids, particularly illudin S, are prominent secondary metabolites in *O. japonicus*.^[^ ^32^ ^]^ Illudin S exhibits potent cytotoxic, antiviral, and antitumor properties, with notable efficacy against multi‐drug resistant tumors.^[^ ^33^ ^]^ Its therapeutic potential has led to the development of irofulven, a novel anticancer drug.^[^ ^34^ ^]^ *P. cornu‐damae*, a poisonous mushroom of the Hypocreaceae family, poses a significant global public health threat due to its potent trichothecenes, a diverse group of chemically related mycotoxins.^[^ ^35^ ^]^ Widely distributed in Japan, China, and East Asia, this mushroom, often called the red deer's horn mushroom, bears a striking resemblance to deer antlers (Figure S4, Supporting Information).^[^ ^36^ ^]^ Tragically, accidental poisonings have occurred due to misidentification with immature *Ganoderma lucidum* and *Cordyceps* medicinal mushrooms.^[^ ^37^ ^]^ Early symptoms of poisoning by *P. cornu‐damae* include vomiting, dehydration, and diarrhea, progressing to more severe symptoms such as anuria, hypotension, and altered consciousness.^[^ ^37^ ^]^ Chemical analysis of toxic metabolites from *P. cornu‐damae* has identified toxic macrocyclic trichothecenes, with roridin E being a major compound demonstrating the most potent cytotoxicity against breast cancer cell lines.^[^ ^29b^ ^]^

**Figure 2:**
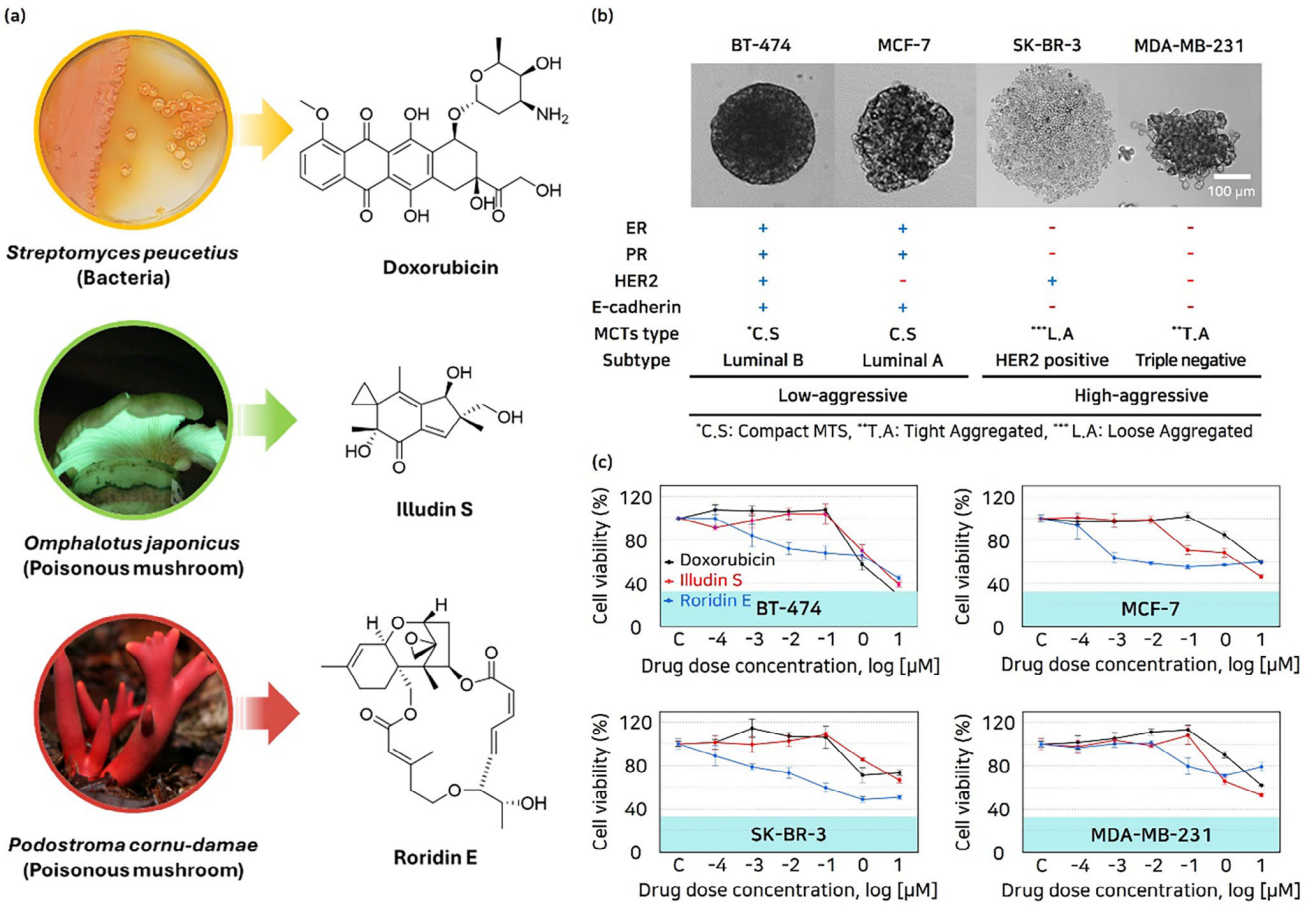
Cytotoxic natural products derived from poisonous mushrooms. A) Chemical structures of doxorubicin, illudin S, and roridin E, along with images of their natural sources. B) Morphology and characteristics of breast MCTs (BT‐474, MCF‐7, SK‐BR‐3, and MDA‐MB‐231). The breast tumor is classified by molecular subtype (ER, PR, and HER2), E‐cadherin expression, and MCTs type. C) Efficacy (viability) of DOX, illudin S, and roridin E on breast tumor monolayer model (BT‐474, MCF‐7, SK‐BR‐3, and MDA‐MB‐231) under 24 h static fluid condition at different drug concentrations.

Fruiting bodies of *O. japonicus* were collected and extracted with 100% methanol, aiming to isolate illudin S as a promising anticancer compound. Similarly, the methanolic extract from potato dextrose agar (PDA) plate cultures of *P. cornu‐damae* was subjected to chemical analysis for the isolation of roridin E as another promising anticancer compound. Liquid chromatography/mass spectrometry (LC/MS)‐guided isolation was employed for both extracts, resulting in the successful isolation of the targeted compounds, illudin S from *O. japonicus* and roridin E from *P. cornu‐damae* (Figures S5,S6, Supporting Information). The structures of illudin S and roridin E were determined through the comparison of their nuclear magnetic resonance (NMR) spectra with previously reported data and LC/MS analysis (**Figure** **2**a; Figures S7,S8, Supporting Information).^[^ ^38^ ^]^ In particular, the natural product roridin E, isolated through this process, was validated to be free from interference by solvents or extraction residues through an efficacy comparison with the negative control compound, trichodermamide A, which was also isolated from *P. cornu‐damae*. This comparison confirmed roridin E as the active compound. (Figure S9, Supporting Information)

Following their isolation and structural identification, we investigated the distinct cytotoxic mechanisms of illudin S and roridin E to elucidate their potential anticancer activities. The cytotoxic pathways induced by illudin S and roridin E differ in their molecular mechanisms. Illudin S primarily induces apoptotic cell death through the formation of DNA adducts, which result in DNA damage, activation of the p53 pathway, and subsequent caspase‐dependent apoptosis.^[^ ^39^ ^]^ Cells deficient in the nucleotide excision repair (NER) pathway are particularly susceptible, consistent with previous findings involving illudin S and its clinical analog, irofulven. Additional mechanisms, such as cell cycle arrest at the G1/S or G2/M checkpoints and the generation of reactive oxygen species (ROS), may further enhance apoptotic signaling.^[^ ^40^ ^]^ In contrast, roridin E exerts its cytotoxic effects by inhibiting ribosomal peptidyl transferase activity, thereby disrupting protein synthesis. This inhibition induces endoplasmic reticulum (ER) stress and activates the unfolded protein response (UPR), which subsequently initiates intrinsic apoptotic pathways involving mitochondrial depolarization and caspase activation.^[^ ^41^ ^]^

The response of the isolated anticancer compounds was evaluated against four breast cancer cell line – BT‐474 (luminal B, IDC), MCF‐7 (luminal A, IDC), SK‐BR‐3 (human epidermal growth factor receptor 2 (HER2) positive, adenocarcinoma), MDA‐MB‐231 (triple negative, adenocarcinoma) – which were classified based on their subtype characteristics, including the expression of estrogen receptor (ER), progesterone receptor (PR), and HER2^[^ ^42^ ^]^ (Figure 2b). These molecular subtypes exhibit distinct prognoses^[^ ^43^ ^]^ and respond differently to chemotherapy,^[^ ^44^ ^]^ making molecular classification a crucial factor in anticancer drug evaluation. Immunohistochemistry (IHC)‐based biomarkers, including ER, PR, and HER2 are widely used as prognostic and predictive markers in breast cancer. These markers play a crucial role in classifying breast cancer subtypes and guiding treatment strategies. The expression status of ER and PR significantly influences cellular proliferation and therapeutic responsiveness. ER+/PR+ tumors generally respond to hormonal signals that regulate cell growth, often exhibiting lower Ki‐67 levels, and are associated with a more favorable prognosis. In contrast, ER−/PR− tumors grow autonomously, are typically characterized by high Ki‐67 expression, and show more aggressive clinical behavior.^[^ ^45^ ^]^ HER2 is a key protein in the epidermal growth factor (EGF) signaling pathway, mediating cell growth and survival. Overexpression of HER2 on the surface of cancer cells leads to abnormally rapid proliferation, and HER2+ tumors are frequently associated with higher recurrence rates and poorer outcomes.^[^ ^46^ ^]^ However, several targeted therapies have been developed against HER2, improving treatment options for this subtype. In contrast, triple‐negative breast cancer, defined by the absence of ER, PR, and HER2 expression, has no available targeted therapies and is treated primarily with cytotoxic chemotherapeutic agents. This subtype is associated with a poorer prognosis, making the evaluation of drug efficacy across different subtypes an important clinical challenge. Cytotoxic chemotherapeutics, which interfere with deoxyribonucleic acid (DNA) or ribonucleic acid (RNA) synthesis and disrupt mitosis during specific phases of the cell cycle, exert their effects by directly damaging DNA. Therefore, their efficacy may vary depending on the proliferation rate of cancer cells. Additionally, targeted therapies function by inducing cell death through the inhibition of specific proteins or enzymes that are overexpressed or aberrantly activated in cancer cells. Therefore, comparative evaluation of drug responses according to HER2 expression is expected to provide valuable insights into the therapeutic potential of targeted agents.

MCTs are characterized based on E‐cadherin expression, which plays a key role in their formation by predominantly promoting the development of compact structures as they evolve.^[^ ^47^ ^]^ Based on these characteristics, MCTs can be classified into compact spheroid (C.S), tight aggregated (T.A), and loose aggregated (L.A) types.^[^ ^16^ ^]^ BT‐474 and MCF‐7 exhibit high levels of E‐cadherin expression, forming the C.S model while SK‐BR‐3 and MDA‐MB‐231 exhibited with no E‐cadherin expression, forming the L.A and T.A model, respectively(Figure 2b).^[^ ^20^, ^48^ ^]^

The efficacy of DOX as a positive control, illudin S, and roridin E was validated using a monolayer model across a concentration range of 10^−4^–10 µm with four types of cell lines. DOX exhibited cytotoxicity at a concentration of 1 µm against tumor cells, progressively decreasing viability in BT‐474 (57.2% to 28.6% from 1 to 10 µm), MCF‐7 (84.8% to 59.4% from 1 to 10 µm), SK‐BR‐3 (73.4% to 71.2% from 1 to 10 µm), and MDA‐MB‐231 (90.1% to 62.1% from 1 to 10 µm) cell lines. Illudin S demonstrated cytotoxicity at a concentration of 1 µm against BT‐474 (69.8% to 38.5% from 1 to 10 µm), SK‐BR‐3 (85.5% to 66.5% from 1 to 10 µm), and MDA‐MB‐231 (65.8% to 52.8% from 1 to 10 µm), and at 0.1 µm against MCF‐7(70.8% to 46.3% from 0.1 to 10 µm), resulting in a progressive decreasing viability cell line. Roridin E efficacy was exhibited at 10^−3^ µm against BT‐474 (83.8% to 44.7% from 10^−3^ to 10 µm), MCF‐7 (63.8% to 55.5% from 10^−3^ to 10 µm), and SK‐BR‐3 (78.8% to 48.9% from 10^−3^ to 10 µm) (Figure 2c).

### 2.3 Validation of Efficacy and Toxicity of Natural Products Using Spheroid Model

**Figure 3:**
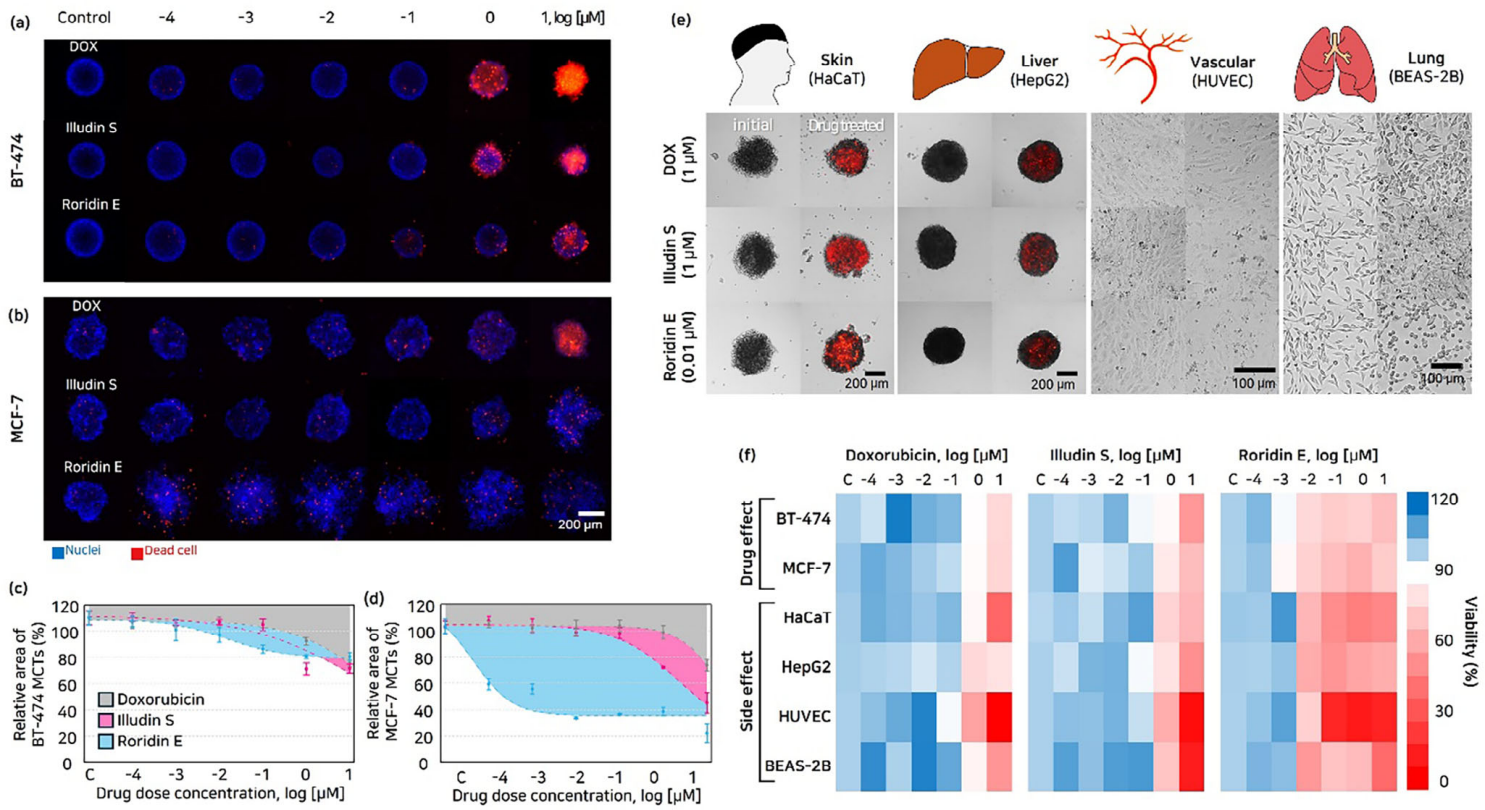
Evaluating the therapeutic range of illudin S and roridin E. A,B) Live/Dead fluorescence staining images of BT‐474 and MCF‐7 MCTs following treatment with DOX, illudin S, or roridin E for 24 h. Nuclei were stained with Hoechst 33342 (blue), and dead cells with propidium iodide (red). C,D) Relative size changes of BT‐474 and MCF‐7 MCTs after 24 h treatment with varying concentrations of DOX, illudin S, and roridin E under static conditions (*n* = 6). E) Cytotoxicity assessment of DOX (1 µM), illudin S (1 µM), and roridin E (0.01 µm) on normal cell lines representing major organs: HepG2 (liver), HaCaT (skin), BEAS‐2B (lung), and HUVECs (vascular endothelium), visualized by propidium iodide staining (*n* = 6). F) Evaluation of drug efficacy and toxicity based on cell viability.

In drug evaluations using MCTs, assessing remission (size and morphological changes) is essential for determining drug efficacy. Cytotoxic chemotherapy typically eliminates rapidly proliferating tumor cells by disrupting the synthesis of DNA and RNA, interfering with mitosis, or directly damaging DNA molecules, leading to a reduction in MCTs size. This approach for analyzing anticancer efficacy is important in therapeutic approaches commonly used for patients, such as adjuvant and neoadjuvant chemotherapy. The relative size change of MCTs serves as a key factor in evaluating remission, as it reflects the inhibition of cell proliferation, apoptosis, and necrosis induced by anti‐cancer drugs. However, it is difficult to reflect that the anticancer drug was accumulated in the intracellular (nucleus and cytoplasm) of the tumor cells.^[^ ^49^ ^]^ These damaged and dying cells remaining on the MCTs surface are analyzed through cell viability, which represents the ratio of live to dead cells. The aggregation model, where the adhesion between adjacent cells is weak, makes it difficult to maintain structure and analyze remission induced by drugs. Therefore, we utilized C.S. MCTs (BT‐474 and MCF‐7), which are of the IDC type, to validate the efficacy of the drugs. The results of drug evaluation conducted on C.S MCTs (BT‐474 and MCF‐7) with drug exposure ranging from 10^−4^ to 10 µm, showed minimum effective concentrations (C_eff,min_) in the MCTs drug efficacy as 1, 1, and 10^−3^ µm for DOX, illudin S, and roridin E, respectively (Figures S10,S11, Supporting Information). A fluorescence‐based viability assay was conducted to visualize cell viability (**Figure** **3**a,b). In BT‐474 MCTs, at C_eff,min_, the evaluation of the relative size change (d_drug_/d_initial_, 110.1%, 92.5%, 71.2%, and 100.0% for Control, DOX, illudin S, and roridin E, *n* = 6) revealed that, when compared to DOX, illudin S was assessed to exhibit the highest drug efficacy (difference between DOX and illudin S, 21.3% for remission). In the case of roridin E, it was evaluated to have less effectiveness in remission compared to DOX (difference between DOX and roridin E, −8.5% for remission) (Figure 3c). In MCF‐7 MCTs, at C_eff,min_, the evaluation of the relative size change (103.1%, 98.7%, 72.1%, and 55.6% for Control, DOX, illudin S, and roridin E, *n* = 6) revealed that, when compared to DOX, illudin S and roridin E were assessed to exhibit high drug efficacy (difference between DOX and illudin S or roridin E, 26.6% and 43.1% for remission, Figure 3d).

Toxicity assessments on organ‐specific cell lines revealed differential toxicity levels, which could guide safer dosage levels and therapeutic index adjustments. Hepatotoxicity due to chemotherapy can include hepatitis, liver fibrosis, and damage to hepatocytes.^[^ ^50^ ^]^ HepG2, which exhibits genotypic features similar to normal liver cells, can be used to screen the hepatotoxicity of new drugs on the liver.^[^ ^51^ ^]^ Skin side effects may include hair follicle damage, oral mucositis, and dermatitis.^[^ ^52^ ^]^ When chemotherapy affects the skin, the rate of cell division in the skin decreases, which reduces the formation of new skin. The epithelial layer is composed of keratinocytes, and HaCaT cells, which are immortalized human dermal keratinocytes, and closely approximate normal keratinocytes.^[^ ^53^ ^]^ They can be used to screen for the cytotoxicity of new drugs on the skin. In the lung, pulmonary toxicities associated with drugs can lead to side effects such as dyspnea, pneumonitis, and bronchitis.^[^ ^54^ ^]^ BEAS‐2B, derived from cancer‐free human bronchial epithelium, is a simian virus 40 large T‐antigen‐immortalized cell line representing the bronchial epithelium, It has been utilized in various in vitro experiment models, such as toxicology assessments, studies on respiratory injury, wound healing, and neoplastic transformation.^[^ ^55^ ^]^ The broad spectrum of cardiovascular complications, cardiotoxicities remains of prime concern, but vascular toxicities have emerged as the second most common group. Consequently, they specifically induce adverse effects such as coronary artery disease, stroke, and vasospasm.^[^ ^56^ ^]^ Human umbilical vein endothelial cell (HUVEC) express many important endothelial markers such as intercellular adhesion molecule‐1, vascular cell adhesion protein 1, and selectins. They have been considered a common model of endothelial cell, as they can respond to physiological and/or pathological stimuli such as high glucose, lipopolysaccharide, and shear stress.^[^ ^57^ ^]^ The human liver is at the centimeter scale^[^ ^58^ ^]^ and the HaCaT cell, which constitutes the epithelial layer, have an in vivo epithelial thickness of 335.59 ± 150.73 µm.^[^ ^59^ ^]^ In HepG2 cells, it has been reported that albumin secretion increases in spheroid models compared to monolayers, suggesting that the spheroid model is more suitable for functional assessments.^[^ ^60^ ^]^ Similarly, in the HaCaT cell line, both proliferation and keratinization occur concurrently in spheroid cultures, enabling more accurate toxicity evaluations. However, without vascular or perusable microchannels, the supply of oxygen and nutrients is limited, leading to the formation of a necrotic core. Therefore, drug evaluation was conducted using spheroids with the largest diameter, where necrosis core formation does not occur, ranging from 300 to 330 µm.^[^ ^8b^ ^]^ However, the normal human bronchial epithelium has an in vivo thickness of ≈28 µm,^[^ ^61^ ^]^ and the endothelium's wall thickness ranges from 0.1 to 10 µm.^[^ ^62^ ^]^ BEAS‐2B cells were used in a monolayer model to assess toxicity in bronchial epithelial cells, as our aim was not to reconstruct airway architecture but rather to reflect the physiological thickness of the normal human bronchial epithelium. For HUVECs, spheroid formation under non‐scaffold conditions led to continuous spontaneous cell death. Based on these observations and considering the inherently limited in vivo thickness of vascular endothelium, a monolayer model was considered more appropriate for vascular toxicity assessment. The evaluation of toxicity using HepG2 (lung, spheroid), HaCaT (skin, spheroid), BEAS‐2B (lung, monolayer), HUVEC (vascular, monolayer), minimum toxic concentrations (C_tox,min_) were determined as 1, 1, and 10^−2^ µm for DOX, illudin S, and roridin E, respectively (Figure 3e). Illudin S has a similar effective concentration range to DOX and achieved the ED_20_ BT‐474 and MCF‐7 (increase of about 3.79‐ and 2.36‐ fold relative to DOX, respectively). Toxicity in skin and vascular was predicted to be similar to that of DOX, but lung and liver toxicity were predicted to be higher. Roridin E has a lower effective concentration range than DOX, with ED_20_ values for BT‐474 and MCF‐7 (increase of about 441.94‐ and 13.88‐fold relative to DOX, respectively). Due to its higher potency, roridin E exhibits a more aggressive cytotoxic profile, making a detailed evaluation of its therapeutic index for potential use as an anticancer agent (Figure 3f and **Table** **1**). Cell viability was assessed using the XTT assay, a colorimetric‐based method that measures cell numbers based on metabolic activity.

**Table 1:** Viability‐based drug efficacy and toxicity.

	ED_20_		IC_80_			
	BT‐474 (3D)	MCF‐7 (3D)	BEAS‐2B (2D)	HUVEC (2D)	HepG2 (3D)	HaCaT (3D)
DOX (µM)	7.01	5.56	2.42	0.46	4.91	1.81
Illudin S (µM)	1.85	2.35	0.72	0.53	1.04	1.99
Roridin E (µM)	7.94 × 10^−2^	1.58 × 10^−2^	1.16 × 10^−2^	8.03 × 10^−3^	3.99 × 10^−3^	5.90 × 10^−3^

### 2.4 Binary T‐MOC Operation and Evaluate the Natural Products Efficacy on Binary T‐MOC

**Figure 4:**
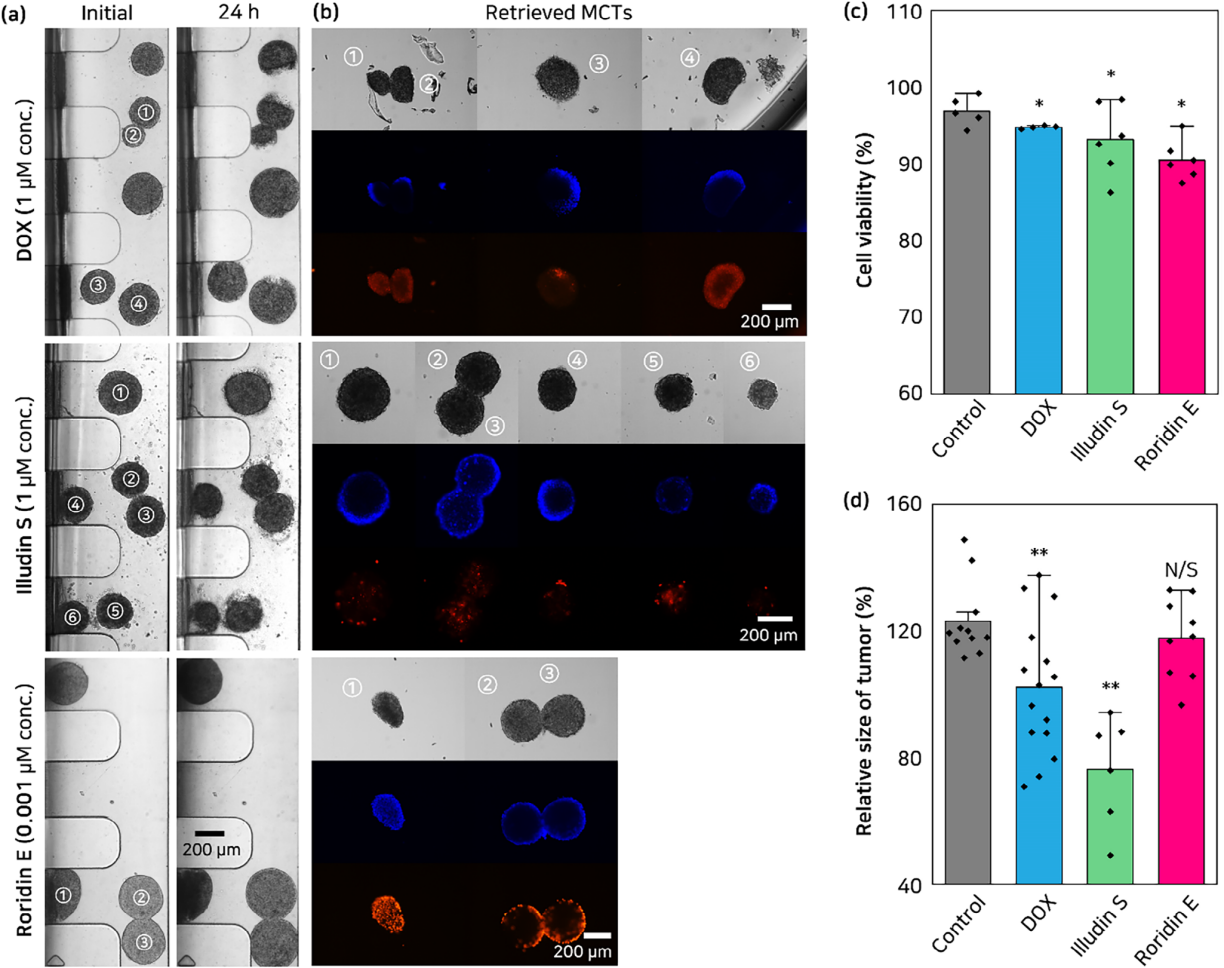
Evaluating the anticancer efficacy of a natural compounds in an alternative model. A,B) Assessment of drug efficacy under 24 h of physiological flow. BT‐474 MCTs were treated with DOX (1 µm), illudin S (1 µm), or roridin E (0.001 µm). Post‐treatment observations revealed MCT proliferation, fusion, cell debris, and signs of drug‐induced remission. C) Live/Dead staining of retrieved MCTs from the tumor compartment following treatment. Nuclei were stained with Hoechst 33342 (blue), and dead cells with propidium iodide (red). Retrieval was enabled by re‐opening the tumor compartment window. D) Quantification of relative size change and cell viability of BT‐474 MCTs before and after drug treatment in the binary T‐MOC. (mean ± SD, ^*^ *p* < 0.05, ^**^ *p* < 0.01).

Drug concentrations used for evaluation in the binary T‐MOC model were determined based on available efficacy and toxicity data. Drug testing using MCTs revealed that DOX and illudin S had similar effective concentration ranges. Notably, the effective concentration of illudin S was comparable to that of irofulven, a clinically evaluated analog.^[^ ^63^ ^]^ In contrast, roridin E exhibited efficacy at a concentration ≈1000 times lower. Roridin E at 0.01 µm demonstrated superior efficacy compared to the clinical concentration of DOX (Figure S12, Supporting Information); however, it exhibited high toxicity in the vascular, liver, lung, and skin tissues. Notably, at this concentration, vascular function could not be maintained, making it unsuitable for application in the binary T‐MOC model. Therefore, in the experiment, DOX, illudin S, and roridin E were used to simulate the in vivo drug effects on the binary T‐MOC mimic IDC model. The concentrations were selected to evaluate the drugs' efficacy while minimizing side effects, with DOX and illudin S at 1 µm and roridin E at 0.001 µm, respectively (**Figure** **4**d). Following drug treatment, IDC samples were retrieved after the reaction for downstream analysis of viability using the live/dead staining assay (Figure 4e). The viability of retrieved samples was quantitatively analyzed by dissociating them into single cells using trypsin‐EDTA for drug efficacy analysis. We confirmed significant anticancer effects on IDC cells (96.8%, 94.7%, 93.2%, and 90.4% for Control, DOX, illudin S, and roridin E, *n* = 3–4, sample = 8–24). Particularly, compared to DOX, illudin S showed lower viability (Figure 4f). The relative size change on IDC MCTs (123.1%, 102.3%, 76.2%, and 117.6% for Control, DOX, illudin S, and roridin E) demonstrated that illudin S had a higher inhibitory effect compared to DOX (with a difference between DOX and illudin S, roridin E of 26.1% and −15.3% for the relative size of the MCTs), In contrast, roridin E did not show a significant tumor size reduction (Figure 4g). The large error bars are attributed to variations in drug delivery and distribution caused by flow differences, depending on the tumor's location within the microchannel. These differences lead to anisotropic remission. While such variability may raise concerns regarding data reliability, it also provides important evidence demonstrating the significant impact of flow dynamics on therapeutic response within the system. Moreover, it suggests that in vivo, therapeutic outcomes may vary substantially depending on vascular proximity and local flow conditions around the tumor. In conclusion, the binary T‐MOC model offers a comprehensive approach for replicating complex in vivo tumor conditions, offering enhanced predictiveness and translational relevance in anticancer drug assessments compared to in vitro NAMs models (Figure S13, Supporting Information). The result of illudin S efficacy indicates relatively high toxicity at elevated concentrations, indicating a narrow therapeutic range that may pose challenges for clinical application. Nonetheless, it exhibited outstanding anti‐cancer effects compared to DOX, with significantly greater efficacy observed in binary T‐MOC models. Roridin E demonstrated an effective drug response at low concentrations, suggesting its potential as an anticancer agent. However, its high toxicity and limited delivery due to vascular barriers, as observed in the T‐MOC model, present a challenge in achieving effective therapeutic concentrations at the tumor site. These limitations suggest that, similar to maytansinoids, roridin E may face challenges as a standalone anticancer agent in clinical applications. However, roridin E can still be utilized as a cytotoxic payload in antibody‐drug conjugate (ADC), where it can be activated upon tumor penetration, even at low doses, to effectively induce cell death. Additionally, its incorporation into drug‐containing nanoparticles could enable targeted delivery and controlled release systems, ensuring effective anticancer activity even at low systemic concentrations. These findings suggest roridin E may be more effective when formulated as drug‐containing nanoparticles or ADC. Moreover, adjuvant therapy is sometimes administered to prevent unexpected metastasis of circulating tumor cells after tumor resection surgery. This implies that anticancer agents can be effective without necessarily requiring trans‐endothelial drug delivery, demonstrating the potential application of roridin E as an adjuvant therapy drug, given its anticancer efficacy at low concentrations and reduced toxicity.

### 2.5 Anisotropic Remission of Natural Products Efficacy in Binary T‐MOC

**Figure 5:**
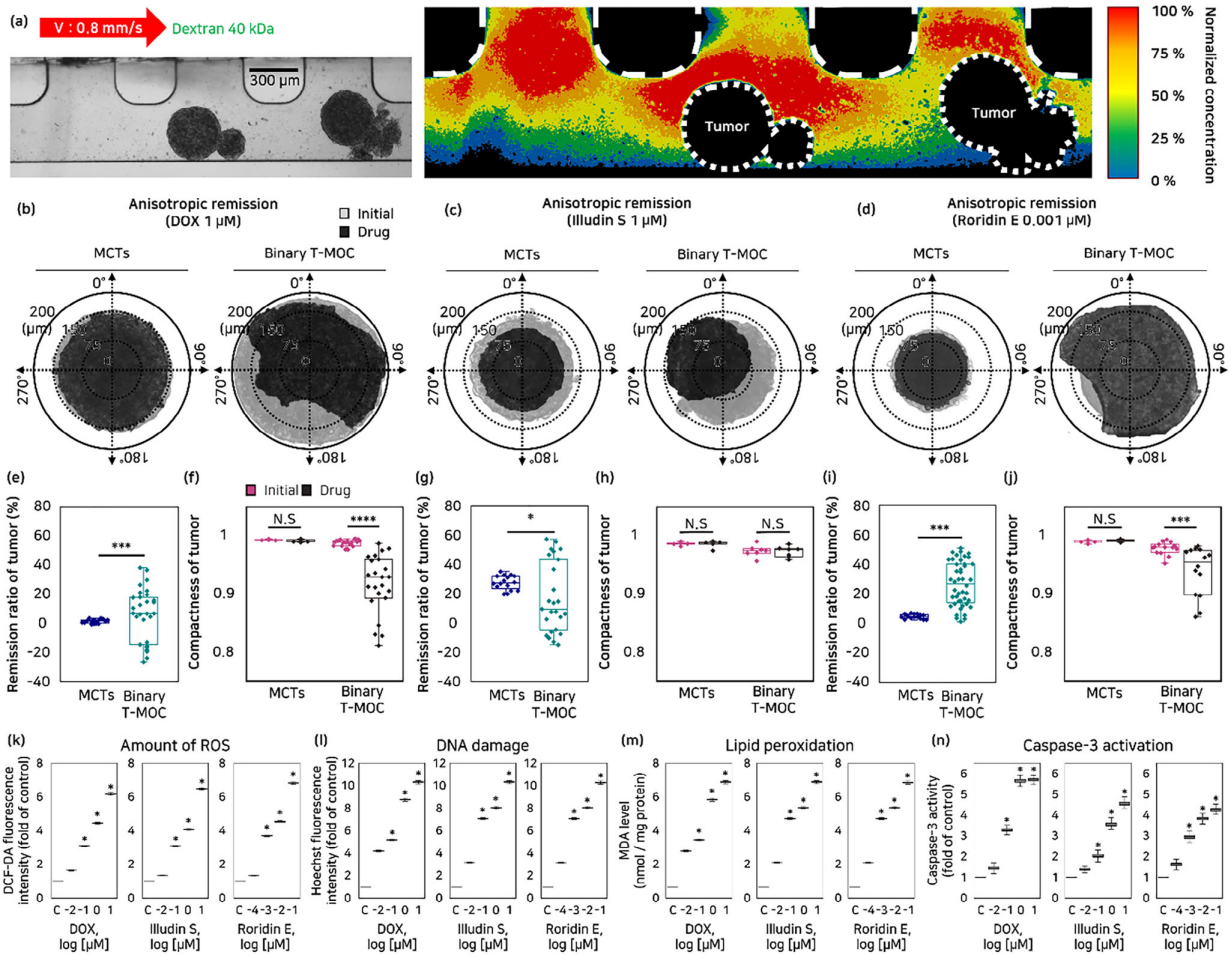
Geometry‐based analysis in binary T‐MOC. A) Distribution and accumulation of FITC‐labeled 40 kDa dextran in the binary T‐MOC under dynamic flow conditions (0.8 mm s^−1^) B–D) Comparison of isotropic remission in the MCTs model (static condition) and anisotropic remission in the binary T‐MOC model (dynamic condition). Geometric‐based analysis of remission ratio E–J) and compactness in the MCTs and binary T‐MOC models following treatment with chemotherapeutic agents—DOX (1 µm), illudin S (1 µm), and roridin E (0.001 µm). The sample sizes for remission ratio analysis were *n* = 5 (DOX), 4 (illudin S), and 5 (roridin E), and for compactness analysis were *n* = 24 (DOX), 8 (illudin S), and 15 (roridin E), respectively. K) Intracellular reactive oxygen species (ROS) generation in BT‐474 cells treated with DOX, illudin S, and roridin E for 24 h. ROS levels were quantified by green fluorescence intensity using H_2_DCFDA staining and are shown as fold changes relative to control. L) Nuclear morphological changes following 24 h treatment with the indicated drugs, assessed by Hoechst 33342 staining. Fold changes in fluorescence intensity were measured by microplate reader. M) Lipid peroxidation levels, assessed by malondialdehyde (MDA) concentrations after drug treatment for 24 h. Control cells received the vehicle only. N) Caspase‐3 activity, determined using a caspase‐3 activity kit after drug treatment for 24 h. (mean ± SD, ^*^ *p* < 0.05, ^***^ *p* < 0.001, ^****^ *p* < 0.0001).

Physiological dynamics and 3D structure of tumor influence drug distribution and accumulation, leading to anisotropic remission and distinct morphological changes in the binary T‐MOC test. To validate drug particle movement within our system, a solution of suspended dextran 40 kDa particles was perfused through the vascular channel using flow dynamics and diffusion. Consequently, tumor and convective forces alter the particle pathway, resulting in distinct drug distribution within the tumor part (**Figure** **5**a). The results of the drug tests using DOX, illudin S, and roridin E with the binary T‐MOC model were compared with those of the MCTs model. In the MCTs model, where drug diffusion occurs primarily through static conditions, isotropic remission was observed alongside uniform morphological changes. In contrast, the binary T‐MOC model, dominated by convective forces, resulted in anisotropic remission, with each drug inducing distinct morphological alterations (Figure 5b–d). Remission was evaluated based on the remission ratio and compactness. For all drugs, there was a difference in the remission ratio between MCTs and binary T‐MOC. While the size decreased uniformly in all directions from the center of mass in MCTs, binary T‐MOC exhibited significant variations depending on the MCTs position and the distribution of the drug (Figure 5e,g,i). The compactness did not show significant changes in the drug tests conducted for all drugs in the MCTs model, and illudin S in the binary T‐MOC models. However, for DOX and roridin E in the binary T‐MOC model, a broad spectrum of compactness values was observed, with a notable decrease due to irregular shapes (engraved, cut‐off shape) (Figure 5f,h,j).

These geometric changes are induced by cell damage mechanisms, primarily through drug accumulation and free radical‐induced damage. To analyze these distinct morphological alterations, levels of reactive oxygen species (ROS), DNA damage, lipid peroxidation, and caspase‐3 activity were measured, showing a general increase in concentration‐dependent manner (Figure 5k–n; Figures S14–S16, Supporting Information). However, the reduction in trans‐endothelial drug concentration and drug accumulation in tumor cells resulted in a complex distribution of drug concentration on the MCTs surface. The trans‐endothelial drug delivery concentration was ≈12% of the intravenous drug concentration.^[^ ^19^ ^]^ Based on the delivered drug concentration, the results showed that illudin S induced more DNA damage than DOX and roridin E at concentrations ≈0.1 µm. DOX exhibited the highest lipid peroxidation activity and caspase‐3 activity, leading to irregular cell morphology. In contrast, at ≈12% of the intravenous concentration of roridin E, drug‐induced damage was not significant, causing cell death only in regions where drug accumulation occurred due to dynamic effects near the MCTs. The remaining areas continued to proliferate, leading to an overall increase in size and changes in MCTs morphology.

The newly established binary T‐MOC platform was employed to evaluate the efficacy of two anticancer natural compounds. By reflecting in vivo biological barriers and dynamics of drug behavior, this platform enabled the observation of their role in drug delivery and tumor remission. Serving as an alternative to animal experiments, it facilitated the determination of therapeutically effective concentrations and the prediction of potential adverse effects, thereby assessing drug efficacy.

## 3 Conclusion

The binary T‐MOC, designed to simulate in vivo‐like drug transport and cellular responses, serves as a platform with enhanced clinical relevance, enabling precise drug screening and dose optimization. This study evaluated the efficacy of anticancer natural products illudin S and roridin E derived from poisonous mushrooms, using a binary T‐MOC that mimics the IDC model. Unlike animal studies, which often require substantial amounts of anticancer agents and make real‐time visualization challenging, the T‐MOC model allows researchers to use smaller quantities of drugs and cells to observe and visualize geometry‐based analysis, drug behavior, and distribution. This approach offers potential applications across various fields, not only evaluating the efficacy of anticancer agents like natural products but also nanoparticle drug delivery systems (DDS), the movement of immune cells from vasculature to tumors, and the efficacy of immunotherapies. This platform demonstrates significant potential to reduce reliance on animal models and improve the accuracy of preclinical drug evaluation, and support ethical and efficient alternatives in drug testing by predicting drug dosage and simulating in vivo effects.

## 4 Experimental Section

### Binary T‐MOC Fabrication

The binary T‐MOC was fabricated using conventional photolithography and soft lithography techniques. SU‐8 3050 photoresist (MicroChem Corp., MA) and SU‐8 developer (MicroChem Corp., MA) were used to create the master mold. Following the fabrication of the master mold, polydimethylsiloxane (PDMS, Dow Corning, MN) was used to produce the system. Patterned PDMS and flat PDMS were bonded using oxygen plasma treatment. The binary T‐MOC was manually cut at the center of the pillar array using a commercially available cutter blade.

The two compartments of the binary T‐MOC were cultured independently to mimic a blood vessel and an MCTs‐laden ECM. For the blood vessel compartment, a pre‐mixed 4 mg mL^−1^ fibrin gel composed of fibrinogen (F8630, Sigma‐Aldrich, MO) and thrombin (T7326, Sigma‐Aldrich, MO) was spread onto the open window channel to form the HUVEC cell attachment bed. To prepare the collagen gel, 10% of 10X PBS and 2.3% sterile 1 N sodium hydroxide (NaOH; DUKSAN, Korea) were added to facilitate collagen polymerization. Rat tail collagen (5 mg mL^−1^; R&D Systems, MN, USA) was used as the stock solution and diluted with sterile distilled water (dH_2_O) to achieve the desired final concentration. All components were kept on ice until use, and all procedures were performed in a biosafety cabinet whenever possible. Polymerization was carried out in a 37 °C, 100% humidity cell incubator for 1 h. After polymerization, a fibronectin solution (0.1 mg mL^−1^) was added to the channel and incubated at 37 °C for 1 h to enhance HUVEC adhesion on the PDMS surface. HUVECs were suspended at a concentration of 7.5 × 10^6^ cells mL^−1^ in HUVECs growth media, and it was injected into the blood vessel channel and incubated vertically for 40 min. The channel was rinsed with HUVECs growth media and incubated in a 37 °C, 5% CO_2_ incubator for one day. To prepare the MCTs‐laden ECM compartment, the channel was coated with dopamine hydrochloride (Sigma‐Aldrich, USA) solution at 2 mg mL^−1^ (10 µm Tris‐HCl [pH8.5]) for overnight in 4 °C refrigerator to promote adhesion between the PDMS surface and ECM. MCTs were suspended in a pre‐mixed collagen solution (4 mg mL^−1^) and injected into the tumor channel. After injection, the tumor compartment was incubated in a 37 °C, 5% CO_2_ incubator for 3 h to allow ECM polymerization. Finally, the tumor compartment was mechanically connected to the fully developed blood vessel compartment using a jig.

### Fungus Materials

Fresh fruiting bodies of *Omphalotus japonicus* were collected from Pocheon, Gyeonggi‐do, Korea, in September 2019. The fungal material was identified through DNA analysis using a modified method.^[^ ^64^ ^]^ Similarly, fresh fruiting bodies of *Podostroma cornu‐damae* were collected from a forest in Pocheon, Korea, in 2020, and mycelium was isolated from the fruiting body tissue. The DNA analysis of mycelium was conducted using a previously described method.^[^ ^64^ ^]^ Polymerase chain reaction (PCR) was performed using fungal‐specific PCR primers ITS1 and ITS4, which target the nuclear ribosomal internal transcribed spacer (ITS) region.^[^ ^65^ ^]^ The amplified ITS region sequences were compared to sequences available in the NCBI Gene Bank. Based on the highest score and homology, the ITS sequences were identified as a match to *O. japonicus* (syn. *Omphalotus guepiniiformis*) and *P. cornu‐damae*, respectively.

### Extraction of Mushrooms and Isolation of Illudin S and Roridin E

The fruiting bodies of *O. japonicus* (1.2 kg) were extracted twice with 100% MeOH (each 8 L × 48 h) at a temperature of 25 °C. The resultant extracts were filtered, and the filtrate was evaporated under reduced pressure using a rotary evaporator to obtain a crude MeOH extract (109.4 g). This crude extract was suspended in distilled water (700 mL) and successively solvent‐partitioned three times with *n*‐hexane (700 mL), dichloromethane (CH_2_Cl_2_, 700 mL), and ethyl acetate (EtOAc, 700 mL), yielding soluble fractions of *n*‐hexane (1.0 g), CH_2_Cl_2_ (1.0 g), and EtOAc (1.1 g). To isolate illudin S, three fractions derived from the solvent partitioning were subjected to LC/MS analysis combined with the reference of an in‐house‐built UV spectra library. This analysis indicated the presence of illudin S in the EtOAc fraction, as it displayed one major peak exhibiting the UV pattern (λ_max_ 240 nm) matching illudin S in our in‐house‐built UV spectra library. Additionally, the major peak showed molecular ion peaks of [M + H]^+^ at *m/z* 265.1 and [M + Na]^+^ at *m/z* 287.1 for illudin S. The EtOAc fraction (1.1 g) was then subjected to preparative HPLC using a gradient solvent system of MeOH/H_2_O (40%‐60% MeOH for 60 min, flow rate of 5 mL/min) to yield five subfractions (subfraction E1‐E5). Through LC/MS‐guided isolation, subfraction E2 (340 mg) was purified by semi‐preparative HPLC with Phenomenex Luna Phenyl Hexyl column (250 × 21.2 mm i.d., 5 µm) using an isocratic system with 28% MeOH/H_2_O (flow rate of 2 mL/min) to afford illudin S (118 mg, *t_R_* = 53.0 min).

Since the amount (0.5 g) of fruiting bodies of *P. cornu‐damae* collected was too small, *P. cornu‐damae* was cultivated on 200 PDA plates (90.0 × 15.0 mm) at 25 °C for 30 days. Once the plates were fully covered with fungal growth, they were fragmented and combined. The consolidated material underwent overnight extraction using 100% MeOH, repeated three times. The resulting MeOH extract was filtered and vacuum‐evaporated, yielding a crude MeOH extract (43.4 g). This extract was dissolved in distilled water (700 mL), and solvent partitioning was performed with *n*‐hexane, CH_2_Cl_2_, EtOAc, and *n*‐butanol. LC/MS analysis identified the EtOAc‐soluble fraction for the isolation of roridin E, as it displayed a peak with the molecular formula of C_29_H_38_O_8_ for roridin E at *m/z* 537.2472 [M + Na]^+^ (calculated for C_29_H_38_O_8_Na, 537.2464) in the quadrupole time‐of‐flight (Q‐TOF) LC/MS analysis. The EtOAc fraction (400 mg) was subjected to C_18_ reversed‐phase open column chromatography (MeOH/H_2_O gradient; 30%–100% MeOH), yielding four subfractions (subfractions E1–E4) based on TLC analysis. Further analysis using Q‐TOF LC/MS identified the peak of roridin E in subfraction E2. This subfraction (29 mg) underwent additional separation using semi‐preparative HPLC with a Phenomenex Luna C_18_ column (250 × 10.0 mm, 5 µm) using an isocratic system with 70% MeOH/H_2_O (flow rate of 2 mL min^−1^), resulting in the isolation of roridin E (6.0 mg, *t_R_* = 36.0 min).

### Cell Preparation for Drug Efficacy and Toxicity Test

Breast tumor cell lines (BT‐474, MCF‐7, SK‐BR‐3, MDA‐MB‐231), HaCaT (skin), HepG2 (liver), BEAS‐2B (lung), and HUVEC (vascular) were utilized to validate drug efficacy and toxicity. Breast tumor cell lines, HaCaT, and HepG2 were cultured in Dulbecco's Modified Eagle's Medium (DMEM)‐high glucose (11965092, GibcoTM, NY), supplemented with 10% FBS (fetal bovine serum, NY), and 1% penicillin/streptomycin (15140122, GibcoTM, NY). The subculture was performed every 3 days or upon reaching 80% confluency. BEAS‐2B cells were cultured in keratinocyte‐SFM and keratinocyte supplements (37000‐015, GibcoTM, NY) containing bovine pituitary extract (13028‐014, GibcoTM, NY) and human recombinant EGF (10450‐013, GibcoTM, NY). HUVECs were cultured in endothelial basal medium supplemented with FBS, hydrocortisone, human fibroblast growth factor, vascular endothelial growth factor, insulin‐like growth factor, ascorbic acid, human Epidermal Growth Factor, antibiotics, and heparin (as per the EGMTM BulletKitTM). All reagents were purchased from Lonza Ltd, Switzerland.

### MCTs Fabrication

Breast MCTs (BT‐474, MCF‐7), HaCaT, and HepG2 spheroids using a droplet‐based microfluidic system previously reported in our work were generated.^[^ ^66^ ^]^ Cell‐encapsulated droplets were generated by suspending cells in cell culture medium and NovecTM 7500 engineered fluid (3MTM, MN, USA), which included 5% surfactant (FluoSurfTM C, FSC‐119, MediSphere Inc., Gyeongsangbuk‐do, Republic of Korea). The cells were then cultured for 2 days in an incubator. Upon completion of MCTs and spheroid production, the droplets were released using 1*H*,1*H*,2*H*,2*H*‐perfluoro‐1‐octanol (370533, Sigma‐Aldrich, MO).

### Monolayer and MCTs Model Preparation for Drug Tests

DOX (doxorubicin hydrochloride, Sigma‐Aldrich, USA), illudin S, and roridin E were dissolved in DMSO and diluted with culture media for control and 10^−4^–10 µm, respectively. Each concentration of drug was applied to both 2D cell monolayers and MCTs for 24 h. For the 2D monolayer test (breast tumor cell line (BT‐474, MCF‐7, SK‐BR‐3, and MDA‐MB‐231), HUVECs, and BEAS‐2B), 1 × 10^5^ cells were seeded and cultured for 12 h to attach to the plate surface. The MCTs (BT‐474, MCF‐7) and spheroid (HepG2, HaCaT) had an average diameter of ≈300 µm and were distributed in each well of the ultra‐low adhesive 96‐well plate (3474, CORNING Inc., ME, USA), with 3 spheroids per well.

### Binary T‐MOC Operation

Maintaining the binary T‐MOC, the culture medium was prepared by mixing DMEM High Glucose and EGM‐2 at a 1:1 ratio. To evaluate the efficacy of the drug, a fresh solution of the drug was perfused through the vascular channel using flow dynamics and hydrostatic pressure. The perfusion velocity was adjusted to 0.8 mm s^−1^ to mimic in vivo capillary blood flow, and it was maintained by a micro‐syringe pump (New Era Pump Systems, Inc., USA) for a constant period of 24 h. Hydrostatic pressure was generated using a reservoir.

### Analysis of the Drug Therapeutics and Particle Distribution Analysis

The remission effect was analyzed using Cellpose 2.0 model^[^ ^67^ ^]^ and Image J software (National Institutes of Health, Bethesda, MD, USA) by comparing the sizes and geometrical features of MCTs before drug treatment and after 24 h. To ensure consistency and reliability in data extraction, a deep learning–based segmentation method, specifically the Cellpose model was employed. Segmentation and contour extraction of MCTs were conducted using Cellpose 2.0, which applies a deep learning algorithm to bright‐field images for accurate cell delineation. The extracted regions of interest (ROI) were subsequently analyzed in ImageJ to evaluate MCTs size distribution, compactness, and remission ratio. Cell viability within monolayer and MCTs was analyzed through the 2,3‐bis‐(2‐methoxy‐4‐nitro‐5‐sulfophenyl)2*H*‐tetrazolium‐5‐carboxanilide (XTT) colorimetric assay (CyQUANTTM XTT Cell Viability assay, X12223, InvitrogenTM, OR, USA). The XTT assay was conducted according to the manufacturer's protocol. MCTs Live/dead staining was conducted with DAPI/PI solution at a ratio of 1:500 for 2 h. The DAPI/PI solution consisted of 4′,6‐diamidino‐2‐phenylindole (DAPI, hoechst 33342, 62249, Thermo Scientific, MA, USA) / propidium iodide (PI, P4864, Sigma‐aldrich, MO, USA) staining. The heatmap imaging for transmittance analysis was conducted using the “Look Up Tables” (LUT) function in the ImageJ software. The LUT represents dark pixels as red and bright pixels as blue.

### Immunofluorescence Assay for Biological Inspection

To evaluate the vascular endothelium and MCTs in the binary organ‐on‐a‐chip system, immunofluorescence staining was conducted. The vascular endothelium was fixed with 4% formaldehyde for 15 min at room temperature, followed by membrane permeabilization using Triton X‐100 (Sigma‐Aldrich, USA) diluted in DPBS for 15 min. Subsequently, Hoechst 33342 (1:200, Thermo Fisher Scientific, USA), anti‐CD31 (1:200, Abcam, UK), and anti‐VE‐cadherin (1:200, Thermo Fisher Scientific, USA) antibodies were introduced into the microchannel and incubated overnight at 37 °C in a 5% CO_2_ atmosphere. After primary antibody binding, samples were incubated with appropriate secondary antibodies for 3 h under the same conditions.

MCTs were fixed with 4% formaldehyde for 15 min at room temperature and permeabilized using Triton X‐100 diluted in DPBS. MCTs were then stained with Hoechst 33342 (1:100), anti‐E‐cadherin (1:100, Abcam, UK), and anti‐Ki‐67 (1:100, Abcam, UK) antibodies, followed by overnight incubation at 37 °C with gentle shaking. After washing with DPBS, secondary antibody staining was performed overnight under the same conditions.

### Remission Ratio Analysis

In chemotherapy, including adjuvant chemotherapy and neoadjuvant chemotherapy, tracking the size and morphology of the target tumor is of significant importance in these therapies. In in vitro drug tests using the MCTs, quantitative analysis of size and morphology changes is also essential to evaluate drug efficacy. The remission ratio, which measures the rate of decrease/increase in length from the initial MCTs centroid, plays a crucial role in this assessment.

(1) $$\mathrm{Remission}\;\mathrm{ratio}=\frac{L_{\mathrm{intial}}-L_{\mathrm{drug}}}{L_{\mathrm{intial}}}$$

where, L_initial_ and L_drug_ are the initial and drug reaction finished sample length from the initial tumor centroid.

### Compactness Analysis

Compactness is utilized as a quantitative factor to assess the morphological changes induced by drugs in MCTs. Compactness is a metric indicating how smooth and close to circular an object is, being proportional to the ratio of an object's area to its perimeter.

(2) $$Compactness=\frac{4*\pi *Area}{Perimeter^{2}}$$

where, area and perimeter are the 2D MCTs's mask area and perimeter.

### Measurement of Intracellular Reactive Oxygen Species

The intracellular reactive oxygen species (ROS) levels were determined using a cell‐permeable fluorescent indicator, 2′,7′‐dichlorofluorescin diacetate (H_2_DCFDA, Sigma‐Aldrich, Saint Louis, MO, USA) or dihydroethidium (DHE, Sigma‐Aldrich, Saint Louis, MO, USA). The cells were plated into black 24‐well flat‐bottom plates and treated with illudin S, roridin E, and DOX, respectively. After 24 h, the cells were loaded with 10 µm H_2_DCFDA or DHE. Following a 30 min incubation in the dark, the non‐reacted H_2_DCFDA or DHE was removed from the plates using phosphate‐buffered saline (PBS). The fluorescence intensity of fluorescent DCF at 495/517 nm (ex/em) or fluorescent DHE at 360/460 nm (ex/em) was measured using a fluorescent microplate reader (Tecan Spark 10 M, Männedorf, Switzerland). Representative cell images were obtained using an IX50 fluorescent microscope (Olympus, Tokyo, Japan) equipped with a CCD camera. Values are expressed as fold increases in fluorescence intensity.

### Measurement of DNA Damage

DNA damage was detected using the DNA‐binding dye hoechst 33342 (Sigma‐Aldrich, Saint Louis, MO, USA). The cells were plated into black 24‐well flat‐bottom plates and treated with illudin S, roridin E, and DOX, respectively. After 24 h, the cells were stained with hoechst 33342 solution (Thermo Fisher Scientific, Waltham, MA, USA) for 10 min. The fluorescence intensity of hoechst 33342 at 350/460 nm (ex/em) was measured using a fluorescent microplate reader (Tecan Spark 10 M, Männedorf, Switzerland). Representative cell images were obtained using an IX50 fluorescent microscope (Olympus, Tokyo, Japan) equipped with a CCD camera. Values are expressed as fold increases in fluorescence intensity.

### Measurement of Lipid Peroxidation

The malondialdehyde (MDA) level was determined using a commercial MDA kit (Abcam, Cambridge, MA, USA). The cells were plated into black 24‐well flat‐bottom plates and treated with illudin S, roridin E, and DOX, respectively. After 24 h, the cells were harvested and lysed in MDA lysis buffer to collect supernatant samples according to the protocol's instructions. The supernatants then reacted with thiobarbituric acid (TBA) reagent to form a fluorometric product. The fluorescence intensity of the TBA adduct formed by the reaction of MDA at 532/553 nm (ex/em) was measured using a fluorescent microplate reader (Tecan Spark 10 M, Männedorf, Switzerland). The MDA levels are expressed as nmol/mg protein.

### Measurement of Caspase‐3 Activity

Caspase‐3 activity was determined using caspase‐3 colorimetric assay kits according to the manufacturer's protocol (BioVison, Milpitas, CA, USA). In brief, the cells were seeded in 100 mm dishes and treated with illudin S, roridin E, and DOX, respectively. The cells were incubated for 24 h and lysed in 50 µL of the lysis buffer supplied in the kit, and incubated on ice for 10 min. Then, 50 µL of 2 × reaction buffer containing 10 mm dithiothreitol and 5 µL of the 4 mm DEVD‐pNA substrate were added to the lysed samples and incubated for 1 h at 37 °C. Subsequently, caspase activity was measured from the absorbance at 400 nm using a microplate reader.

### Statistical Analysis

Statistical analysis was conducted using analysis of variance (ANOVA) followed by a multiple comparison test with a Bonferroni adjustment. The analysis was carried out using SPSS version. 19.0 (SPSS Inc., Chicago, IL, USA). All the assays were performed in triplicate and repeated at least three times. Data with P values of less than 0.05 were considered statistically significant.

## Conflict of Interest

The authors declare no conflict of interest.

## Supporting information



Supporting Information

Supplemental Video 1

## Data Availability

The data that support the findings of this study are available from the corresponding author upon reasonable request.

## References

[1] F. F. Hefti , BMC Neurosci. 2008, 9, 1. 19091004 10.1186/1471-2202-9-S3-S7 PMC2604885

[2] H. Wang , P. C. Brown , E. C. Chow , L. Ewart , S. S. Ferguson , S. Fitzpatrick , B. S. Freedman , G. L. Guo , W. Hedrich , S. Heyward , Clin. Transl. Sci. 2021, 14, 1659. 33982436 10.1111/cts.13066 PMC8504835

[3] A. Akhtar , Camb. Q. Healthc. Ethics 2015, 24, 407. 26364776 10.1017/S0963180115000079 PMC4594046

[4] S. K. Doke , S. C. Dhawale , Saudi Pharm. J. 2015, 23, 223. 26106269 10.1016/j.jsps.2013.11.002 PMC4475840

[5] P.‐J. Zushin , S. Mukherjee , J. C. Wu , Am Soc. Clin. Investig. 2023, 133, 175824. 10.1172/JCI175824 PMC10617761 37909337

[6] a) S. Lee , H.‐I. Jung , J. Lee , Y. Kim , J. Chung , H. S. Kim , J. Lim , K. C. Nam , Y.‐S. Lim , H. S. Choi , Lab Chip 2024, 24, 3243. 38836406 10.1039/d4lc00249k

[7] a) X. Chen , R. Roberts , Z. Liu , W. Tong , Nat. Commun. 2023, 14, 7141. 37932302 10.1038/s41467-023-42933-9 PMC10628291

[8] a) S. Y. Park , H. J. Hong , H. J. Lee , BioChip J. 2023, 17, 24;

[9] a) G. Jin , D. Kim , S. Mun , S. Bang , BioChip J. 2024, 18, 186.

[10] S. Y. Choi , M. Kim , S. J. Kang , Y. W. Choi , S. Maeng , S.‐H. Kim , I. H. Chang , BioChip J. 2023, 17, 496.

[11] A. G. Atanasov , S. B. Zotchev , V. M. Dirsch , C. T. Supuran , Nat. Rev. Drug Discovery 2021, 20, 200. 33510482 10.1038/s41573-020-00114-z PMC7841765

[12] T. Ling , W. H. Lang , J. Maier , M. Quintana Centurion , F. Rivas , Molecules 2019, 24, 2012. 31130671 10.3390/molecules24102012 PMC6571673

[13] A. M. Hasan‐Abad , A. Atapour , A. Sobhani‐Nasab , H. Motedayyen , R. ArefNezhad , Cancer Reports 2024, 7, 70012. 10.1002/cnr2.70012 PMC11506041 39453820

[14] J. Chen , X. Wang , T. Xia , Y. Bi , B. Liu , J. Fu , R. Zhu , Biomed. Pharmacother. 2021, 142, 111927. 34339914 10.1016/j.biopha.2021.111927

[15] a) G. Francia , S. Man , B. Teicher , L. Grasso , R. S. Kerbel , Mol. Cell. Biol. 2004, 24, 6837; 15254249 10.1128/MCB.24.15.6837-6849.2004 PMC444854

[16] S. J. Han , S. Kwon , K. S. Kim , Cancer Cell Int. 2021, 21, 152. 33663530 10.1186/s12935-021-01853-8 PMC7934264

[17] a) A. S. Nunes , A. S. Barros , E. C. Costa , A. F. Moreira , I. J. Correia , Biotechnol. Bioeng. 2019, 116, 206; 30367820 10.1002/bit.26845

[18] a) M. Hockel , P. Vaupel , J. Nat. Cancer Instit. 2001, 93, 266; 10.1093/jnci/93.4.266 11181773

[19] Y. Kim , J. Lee , S. Lee , H. I. Jung , B. Kwak , Biosens. Bioelectron. 2024, 243, 115787. 10.1016/j.bios.2023.115787 39492183

[20] a) M. Wu , H. B. Frieboes , S. R. McDougall , M. A. Chaplain , V. Cristini , J. Lowengrub , J. Theor. Biol. 2013, 320, 131; 23220211 10.1016/j.jtbi.2012.11.031 PMC3576147

[21] M. Welter , H. Rieger , PLoS One 2013, 8, 70395. 10.1371/journal.pone.0070395 PMC3734291 23940570

[22] J. M. Munson , A. C. Shieh , Cancer Manage. Res. 2014, 317. 10.2147/CMAR.S65444 PMC4144982 25170280

[23] a) A. M. Pollet , J. M. den Toonder , Bioengineering‐Basel 2020, 7, 17. 32085464 10.3390/bioengineering7010017 PMC7175276

[24] C. H. Heldin , K. Rubin , K. Pietras , A. Östman , Nat. Rev. Cancer 2004, 4, 806. 15510161 10.1038/nrc1456

[25] X.‐Y. Ke , V. W. Ng , S.‐J. Gao , Y. W. Tong , J. L. Hedrick , Y. Y. Yang , Biomaterials 2014, 35, 1096. 24183698 10.1016/j.biomaterials.2013.10.049

[26] Y. Harahap , P. Ardiningsih , A. Corintias Winarti , D. J. Purwanto , Drug Des. Devel. Ther. 2020, 14, 3469. 10.2147/DDDT.S251144 PMC7457744 32921983

[27] a) G. Venturella , V. Ferraro , F. Cirlincione , M. L. Gargano , Int. J. Mol. Sci. 2021, 22, 634; 33435246 10.3390/ijms22020634 PMC7826851

[28] S. Lee , M. Jang , R. Ryoo , J. Roh , S.‐K. Ko , K. H. Kim , Arch. Pharmacal Res. 2024, 47, 272. 10.1007/s12272-024-01486-1 38416389

[29] a) J. S. Yu , S. Y. Jeong , C. Li , T. Oh , M. Kwon , J. S. Ahn , S.‐K. Ko , Y.‐J. Ko , S. Cao , K. H. Kim , Arch. Pharmacal Res. 2022, 45, 105; 10.1007/s12272-022-01372-8 35201589

[30] S. Aoki , T. Aboshi , T. Onodera , K.‐i. Kimura , D. Arai , Y. Iizuka , T. Murayama , Biotechnol. Biochem. 2021, 85, 1364. 10.1093/bbb/zbab063 33851984

[31] M. J. Ting , C. L. Lin , H. L. Chen , S. F. Chou , F. S. Jaw , K. C. Tsai , C. C. Hsieh , Hong Kong J. Emerg. Med. 2024, 31, 472.

[32] Y. Uto , K. Sasaki , M. Takahashi , K. Morimoto , K. Inoue , Anal. Sci. 2019, 35, 789. 30930353 10.2116/analsci.19P053

[33] M. J. Kelner , T. C. McMorris , W. T. Beck , J. M. Zamora , R. Taetle , Cancer Res. 1987, 47, 3186. 3472654

[34] M. J. Kelner , T. C. McMorris , L. Estes , M. Rutherford , M. Montoya , J. Goldstein , K. Samson , R. Starr , R. Taetle , Biochem. Pharmacol. 1994, 48, 403. 8053936 10.1016/0006-2952(94)90113-9

[35] H. N. Kim , H. H. Do , J. S. Seo , H. Y. Kim , Clin Exp Emerg Med. 2016, 3, 186. 27752639 10.15441/ceem.15.028 PMC5065333

[36] K. Gonmori , H. Fujita , K. Yokoyama , K. Watanabe , O. Suzuki , Forensic Toxicol. 2011, 29, 85.

[37] K. Yokoyama , K. Gonmori , Chudoku Kenkyu: Chudoku Kenkyukai jun Kikanshi = Japan. J. Toxicol. 2009, 22, 240. 19882971

[38] a) Y. Saikawa , H. Okamoto , T. Inui , M. Makabe , T. Okuno , T. Suda , K. Hashimoto , M. Nakata , Tetrahedron 2001, 57, 8277.

[39] Y. Lee , Y. Y. Lee , J. Park , A. Maksakova , D. Seo , J. Kim , J. E. Yeom , Y. Kim , C.‐H. Kim , R. Ryoo , Biomed. Pharmacother. 2025, 182, 117795. 39740390 10.1016/j.biopha.2024.117795

[40] N. G. Jaspers , A. Raams , M. J. Kelner , J. M. Ng , Y. M. Yamashita , S. Takeda , T. C. McMorris , J. H. Hoeijmakers , DNA Repair 2002, 1, 1027 12531012 10.1016/s1568-7864(02)00166-0

[41] Y. Li , D. Liu , Z. Cheng , P. Proksch , W. Lin , RSC Adv. 2017, 7, 7259.

[42] S. Lee , C. J. Woo , H.‐I. Jung , K. C. Nam , J. S. Lim , B. S. Kwak , ACS Biomater. Sci. Eng. 2024, 10, 2477. 38483467 10.1021/acsbiomaterials.4c00005

[43] A. Prat , C. Fan , A. Fernández , K. A. Hoadley , R. Martinello , M. Vidal , M. Viladot , E. Pineda , A. Arance , M. Muñoz , BMC Med. 2015, 13, 1. 26684470 10.1186/s12916-015-0540-z PMC4683815

[44] R. Rouzier , C. M. Perou , W. F. Symmans , N. Ibrahim , M. Cristofanilli , K. Anderson , K. R. Hess , J. Stec , M. Ayers , P. Wagner , Clin. Cancer Res. 2005, 11, 5678. 16115903 10.1158/1078-0432.CCR-04-2421

[45] M. R. Yoon , J. Y. Rhu , B. J. Song , B. J. Chae , T.‐K. Yoo , J. Breast Dis. 2019, 7, 1.

[46] a) T. Nakada , K. Sugihara , T. Jikoh , Y. Abe , T. Agatsuma , Chem. Pharm. Bull. 2019, 67, 173; 10.1248/cpb.c18-00744 30827997

[47] R.‐Z. Lin , L.‐F. Chou , C.‐C. M. Chien , H.‐Y. Chang , Cell Tissue Res. 2006, 324, 411. 16489443 10.1007/s00441-005-0148-2

[48] a) K. M. Hajra , D. Y. Chen , E. R. Fearon , Cancer Res. 2002, 62, 1613. 11912130

[49] O. Tacar , P. Sriamornsak , C. R. Dass , J. Pharm. Pharmacol. 2013, 65, 157. 23278683 10.1111/j.2042-7158.2012.01567.x

[50] F. Vernuccio , M. Dioguardi Burgio , F. Barbiera , S. Cusmà , G. Badalamenti , M. Midiri , V. Vilgrain , G. Brancatelli , Abdom. Radiol. 2019, 44, 3312. 10.1007/s00261-019-02193-y 31435760

[51] H. Gerets , K. Tilmant , B. Gerin , H. Chanteux , B. Depelchin , S. Dhalluin , F. Atienzar , Cell Biol. Toxicol. 2012, 28, 69. 22258563 10.1007/s10565-011-9208-4 PMC3303072

[52] G. Fabbrocini , N. Cameli , M. C. Romano , M. Mariano , L. Panariello , D. Bianca , G. Monfrecola , J. Experiment. Clin. Cancer Res. 2012, 31, 1. 10.1186/1756-9966-31-50 PMC3583303 22640460

[53] P. Boukamp , R. T. Petrussevska , D. Breitkreutz , J. Hornung , A. Markham , N. E. Fusenig , J. Cell Biol. 1988, 106, 761. 2450098 10.1083/jcb.106.3.761 PMC2115116

[54] L. Li , H. Mok , P. Jhaveri , M. D. Bonnen , A. G. Sikora , N. T. Eissa , R. U. Komaki , Y. T. Ghebre , Expert Rev. Anticancer Ther. 2018, 18, 1041. 29996062 10.1080/14737140.2018.1500180 PMC6290681

[55] F. Zhao , W. T. Klimecki , J. Appl. Toxicol. 2015, 35, 945. 25524072 10.1002/jat.3094 PMC4474793

[56] J. Herrmann , Nat. Rev. Cardiol. 2020, 17, 503. 32218531 10.1038/s41569-020-0347-2 PMC8782612

[57] Y. Cao , Y. Gong , L. Liu , Y. Zhou , X. Fang , C. Zhang , Y. Li , J. Li , J. Appl. Toxicol. 2017, 37, 1359. 28383141 10.1002/jat.3470

[58] L. Sun , L. Hui , J. Molecul. Cell Biol. 2020, 12, 607. 10.1093/jmcb/mjaa013 PMC7683012 32236564

[59] D. Di Stasio , D. Lauritano , H. Iquebal , A. Romano , E. Gentile , A. Lucchese , Diagnostics 2019, 9, 90. 31390841 10.3390/diagnostics9030090 PMC6787684

[60] S. C. Ramaiahgari , M. W. Den Braver , B. Herpers , V. Terpstra , J. N. Commandeur , B. van de Water , L. S. Price , Arch. Toxicol. 2014, 88, 1083. 24599296 10.1007/s00204-014-1215-9

[61] C. Leung , S. J. Wadsworth , S. J. Yang , D. R. Dorscheid , AJP‐Lung Cell. Mol. Physiol. 2020, 318 , L1063. 10.1152/ajplung.00190.2019 32208929

[62] M. Félétou , in Colloquium Series on Integrated Systems Physiology: From Molecule to Function to Disease, (Eds.: D.N. Granger , J.P. Granger ), Morgan & Claypool Life Sciences, San Rafael, California 2011, 1.

[63] S. G. Eckhardt , S. D. Baker , C. D. Britten , M. Hidalgo , L. Siu , L. A. Hammond , M. A. Villalona‐Calero , S. Felton , R. Drengler , J. G. Kuhn , J. Clin. Oncol. 2000, 18, 4086. 11118470 10.1200/JCO.2000.18.24.4086

[64] L. SB , PCR Prot.: A Guid. Meth. Appl. 1990, 282.

[65] M. Gardes , T. D. Bruns , Mol. Ecol. 1993, 2, 113. 8180733 10.1111/j.1365-294x.1993.tb00005.x

[66] B. Kwak , Y. Lee , J. Lee , S. Lee , J. Lim , J. Controlled Release 2018, 275, 201. 10.1016/j.jconrel.2018.02.029 29474963

[67] C. Stringer , T. Wang , M. Michaelos , M. Pachitariu , Nat. Methods 2021, 18, 100. 33318659 10.1038/s41592-020-01018-x

