# Supplementary material for: Alternative Models for Anticancer Drug Discovery From Natural Products Using Binary Tumor‐Microenvironment‐on‐a‐Chip
Source: Adv Sci (Weinh). 2025 Jul 28;12(40):e07944. doi: 10.1002/advs.202507944 (PMC12561429; doi:10.1002/advs.202507944)
Supplement: Supplementary file 1 — Supporting Information [file ADVS-12-e07944-s001.docx]

**Supporting information**

**Alternative Models for Anticancer Drug Discovery from Natural Products using Binary Tumor-Microenvironment-on-a-Chip**

Youngwon Kim ^a,b,1^, Si Hyeon Chae ^c,1^, Dahae Lee ^d,1^, Bum Soo Lee ^c^, Jiseok Lim ^e,f^, Hyo-Il Jung ^a,g^, Ki Hyun Kim ^c,*^, and Bong Seop Kwak ^b,f**^

^a^School of Mechanical Engineering, Yonsei University, 50 Yonsei-ro, Seodaemun-gu, Seoul, 13722, Republic of Korea

^b^College of Medicine, Dongguk University, 32 Dongguk-ro, Ilsandong-gu, Goyangsi, Gyeonggi-do, 10326, Republic of Korea

^c^School of Pharmacy, Sungkyunkwan University, Suwon, 16419, Republic of Korea

^d^College of Korean Medicine, Gachon University, Seongnam 13120, Republic of Korea

^e^School of Mechanical Engineering, Yeungnam University, 280 Daehak-ro, Gyeongsan-si, Gyeongsangbuk-do, 38541, Republic of Korea

^f^MediSphere Inc., 280, Daehak-ro, Gyeongsan-si, Gyeongsangbuk-do, 38541, Republic of Korea

^g^The DABOM Inc., 50 Yonsei-ro, Seodaemun-gu, Seoul, 03722, Republic of Korea

*Co-Corresponding author

E-mail address: khkim83@skku.edu

Telephone number: +82-31-290-7700

Mobile-phone number: +82-10-8523-3265

**Corresponding author

E-mail address: bskwak82@gmail.com

Telephone number: +82-31-961-5803

Mobile-phone number: +82-10-4477-3865

^1^ These authors contributed equally to this paper.

**General experimental procedure for isolation and structural elucidation of natural products.** Optical rotation was measured using a Jasco P-1020 polarimeter (Jasco, Easton, MD, USA). Infrared (IR) spectrum was recorded on a Bruker IFS-66/S FT-IR spectrometer (Bruker, Karlsruhe, Germany). Ultraviolet (UV) spectrum was acquired on an Agilent 8453 UV-visible spectrophotometer (Agilent Technologies, Santa Clara, CA, USA). High-resolution (HR)-electrospray ionization (ESI) mass data were recorded on an Agilent 1290 Infinity II series with a 6545 LC/Q-TOF mass spectrometer (Agilent Technologies). NMR spectra were recorded on a Bruker AVANCE III spectrometer (Bruker). Preparative high-performance liquid chromatography (HPLC) was conducted using a Waters 1525 binary HPLC pump with a Waters 996 photodiode array detector (Waters) and an Agilent Eclipse C_18_ column (250 × 21.2 mm, 5 μm; flow rate: 5 mL/min) (Agilent Technologies), and semi-preparative HPLC was performed using a Shimadzu Prominence HPLC System with SPD-20A/20AV Series Prominence HPLC UV-Vis Detectors (Shimadzu, Tokyo, Japan). LC/MS analysis was performed on an Agilent 1200 series HPLC system with a diode array detector and 6130 Series ESI mass spectrometer using an analytical Kinetex C18 100 Å column (100 mm × 2.1 mm i.d., 5 μm) (Phenomenex, Torrance, CA). Column chromatography was performed using silica gel 60 (Merck, Darmstadt, Germany, 230-400 mesh) and RP-C_18_ silica gel (Merck, 230-400 mesh). Thin-layer chromatography (TLC) was conducted using precoated silica gel F_254_ plates and reverse-phase (RP)-18 F_254s_ plates (Merck). Spots on TLC were detected using UV light and heating after dipping in anisaldehyde-sulfuric acid.

**
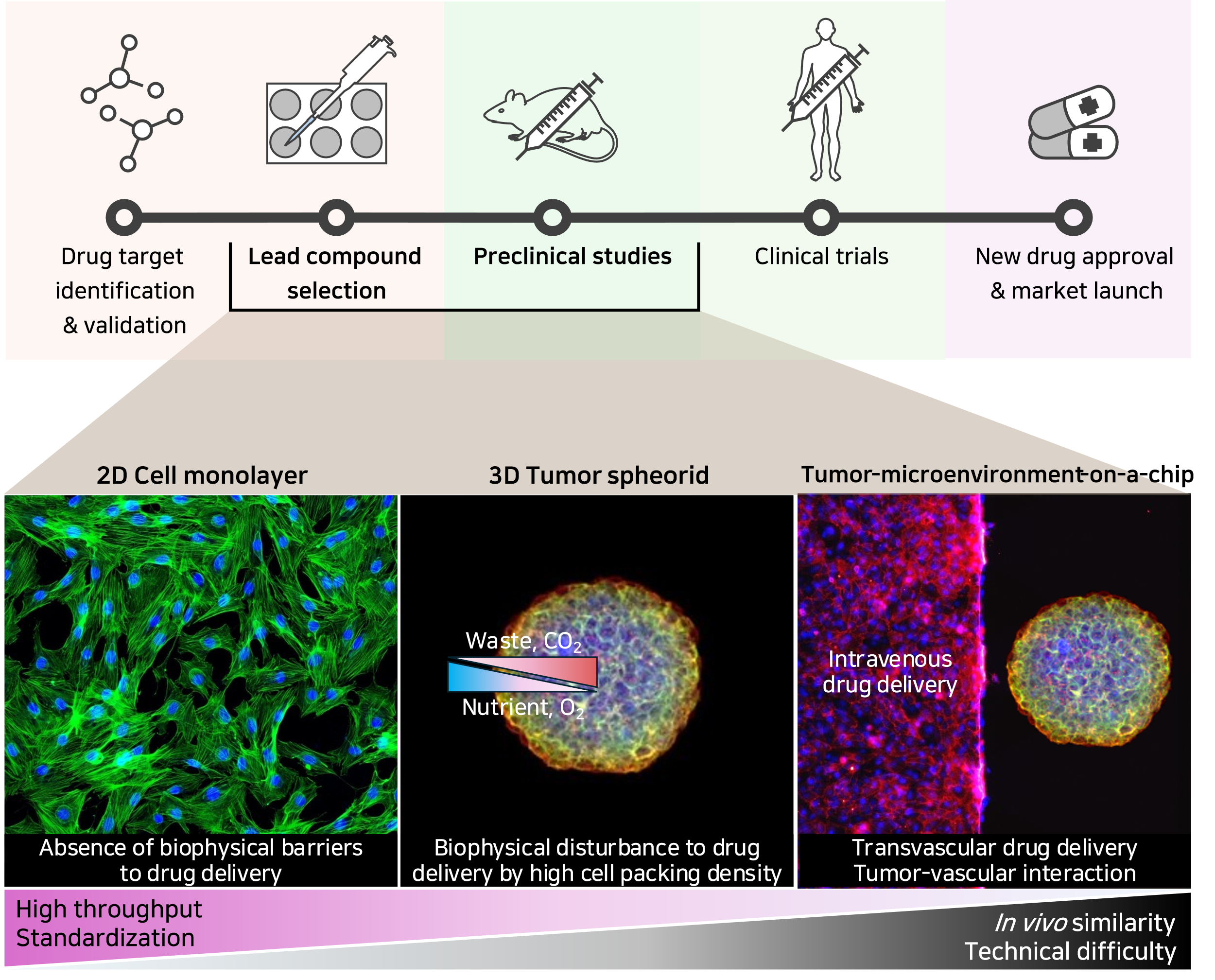
**

**Figure S1. The role of in vitro models in drug development process and their characteristics.** Conceptual comparison of three in vitro drug testing platforms: 2D monolayer, multicellular tumor spheroids (MCTs), and tumor microenvironment-on-a-chip (T-MOC) systems. The 2D model offers high productivity but lacks physiological relevance. The MCTs model introduces biophysical barriers and spatial gradients, partially mimicking tumor microenvironments. The T-MOC system incorporates vascular channels and ECM components, enabling simulation of in vivo drug resistance mechanisms.

**
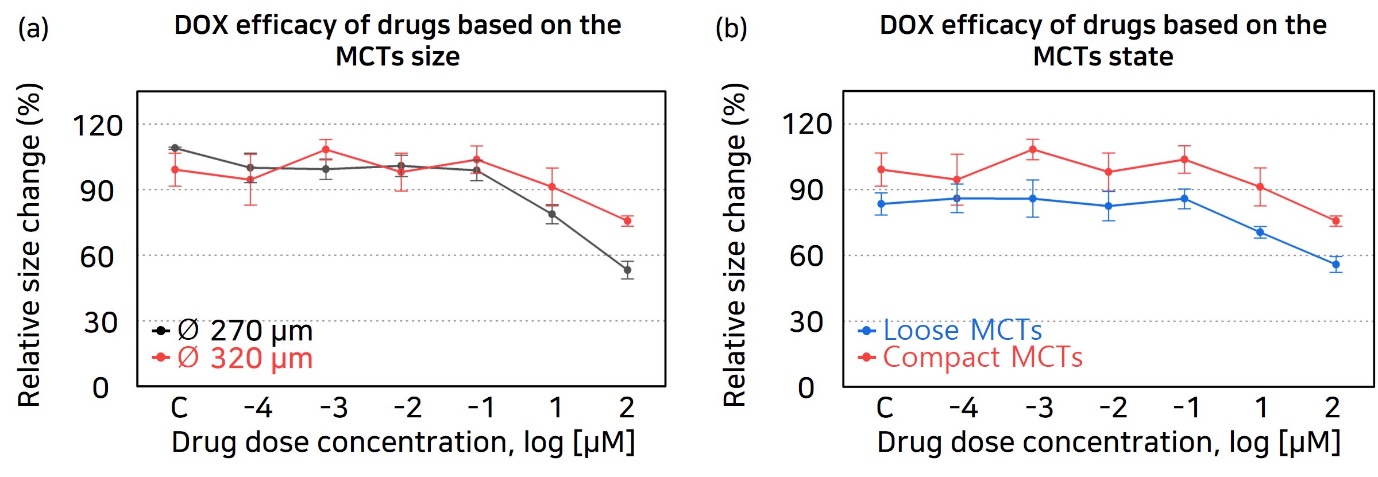
Figure S2. The variations in drug efficacy due to differences in MCTs size and development. a**, The relative size changes according to the difference in MCTs size. Fully developed BT-474 MCTs (initial average diameter: 270 μm and 320 μm) were treated with DOX ranging from 10^-4^ μM to 10 μM for 24 h. As the size of MCTs increases, we can observe an increase in drug resistance. **b**, The relative size changes according to the difference in MCTs development. Initial average diameter 320 μm BT-474 MCTs (loose MCTs group and compact MCTs group) were treated with DOX ranging from 10^-4^ μM to 10 μM for 24 h. It is difficult to distinguish whether the decrease in size is due to the drug or is a result of compaction during the spheroid development process.

**
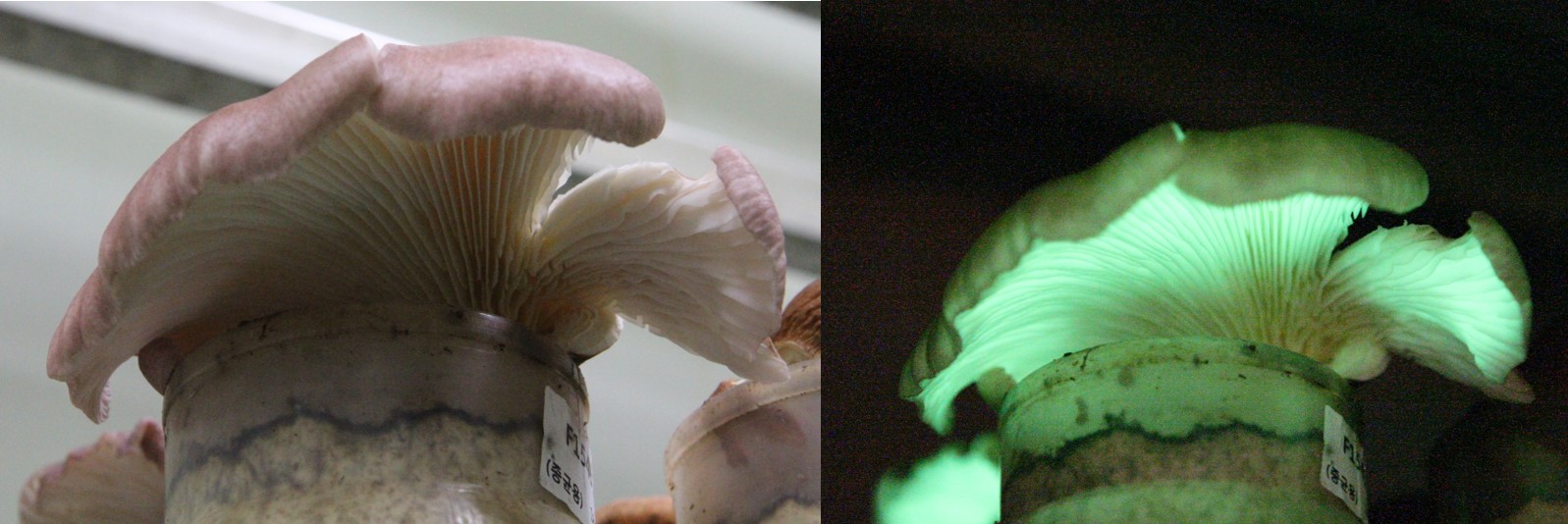
**

**Figure S3. The photos of *O. japonicus* (left) and bioluminescent *O. japonicus* in the dark (right).**


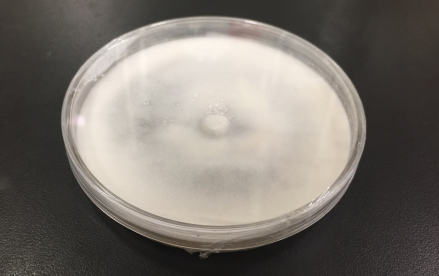

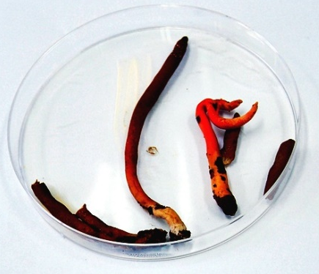


**Figure S4. The photos of *P. cornu-damae* (left) and a plate culture of the fungus *P. cornu-damae* (right).**


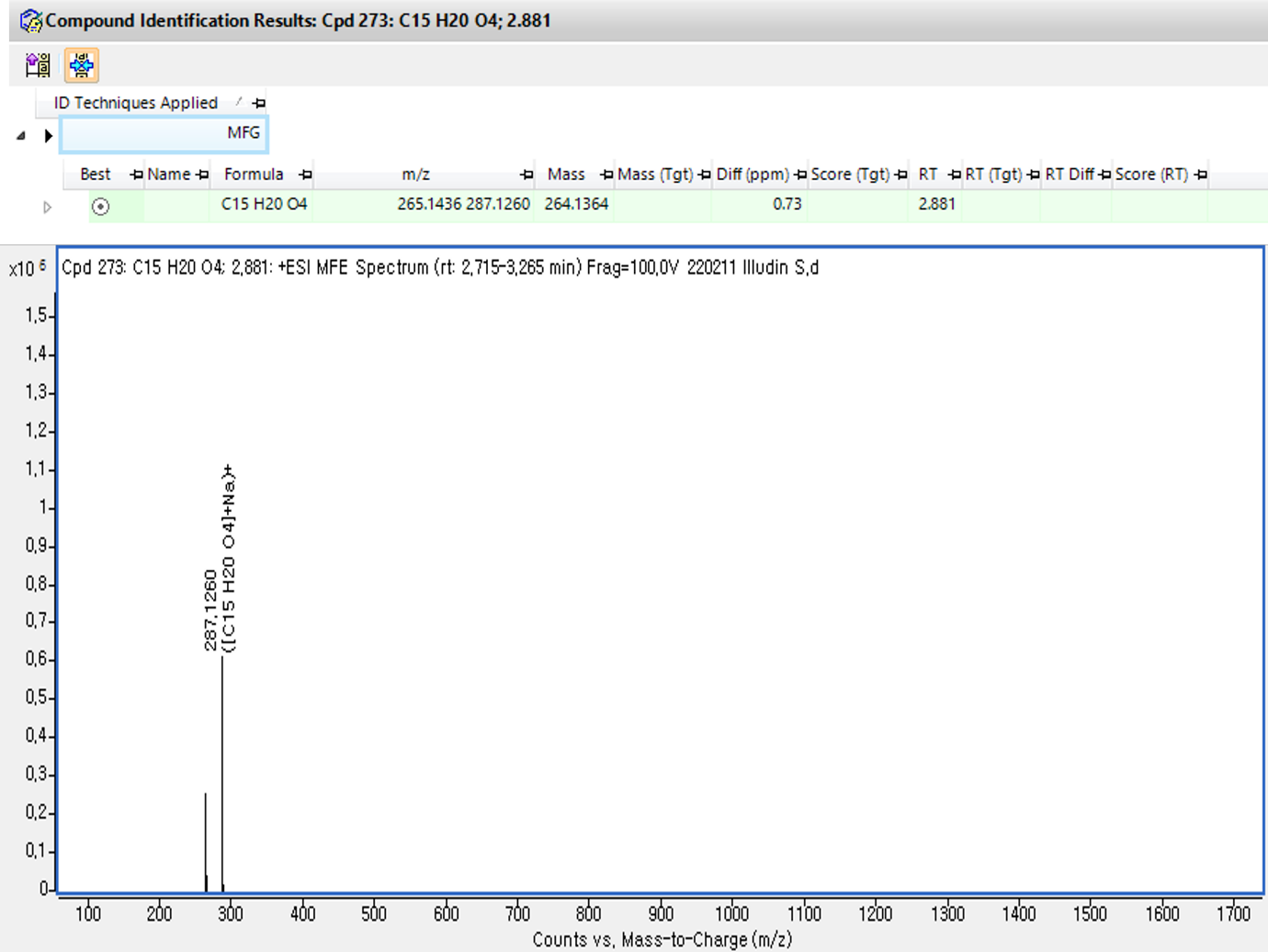


**Figure S5. HR-ESIMS data of illudin S.**


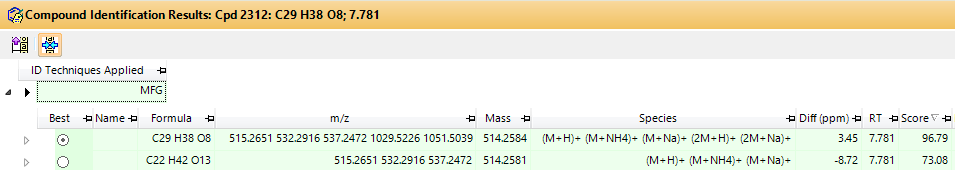


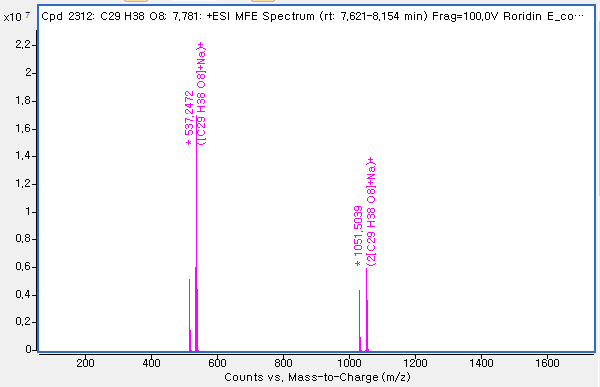


**Figure S6. HR-ESIMS data of roridin E.**


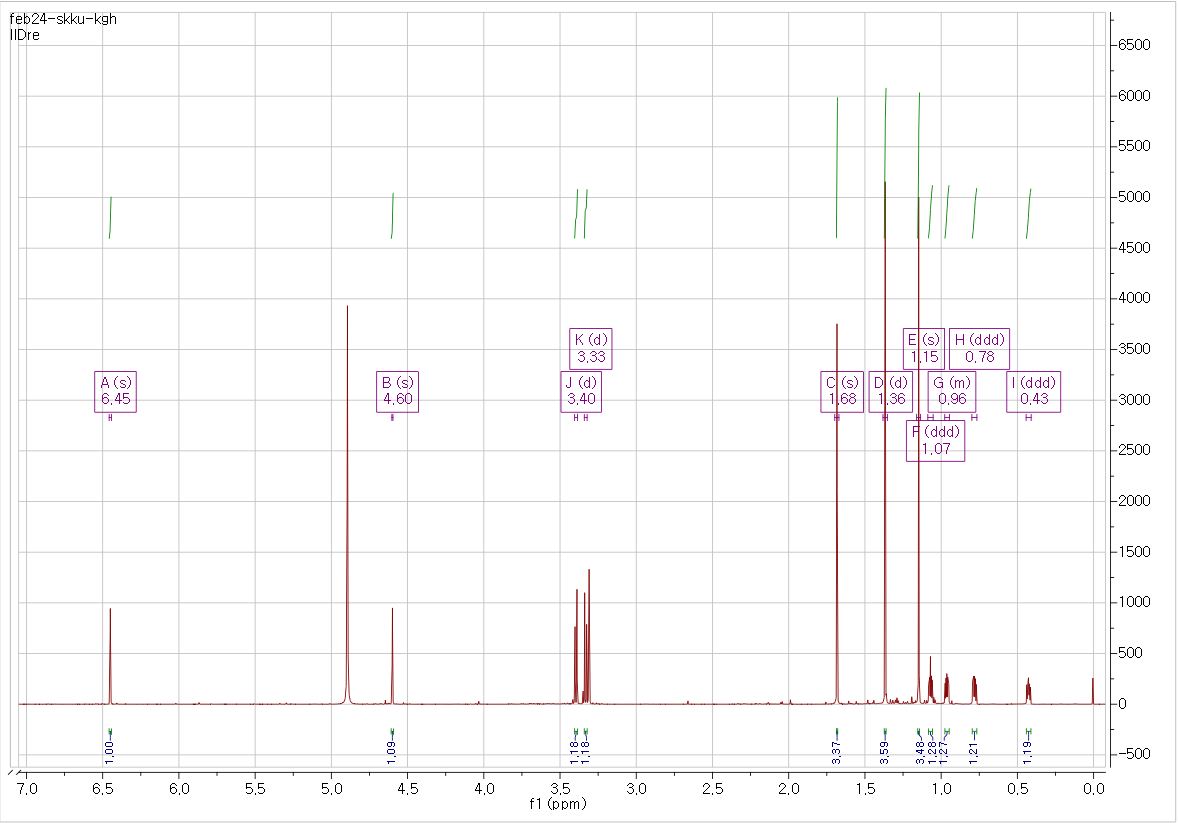


**Figure S7. The ^1^H NMR spectrum of illudin S (CD_3_OD, 850 MHz).**

**
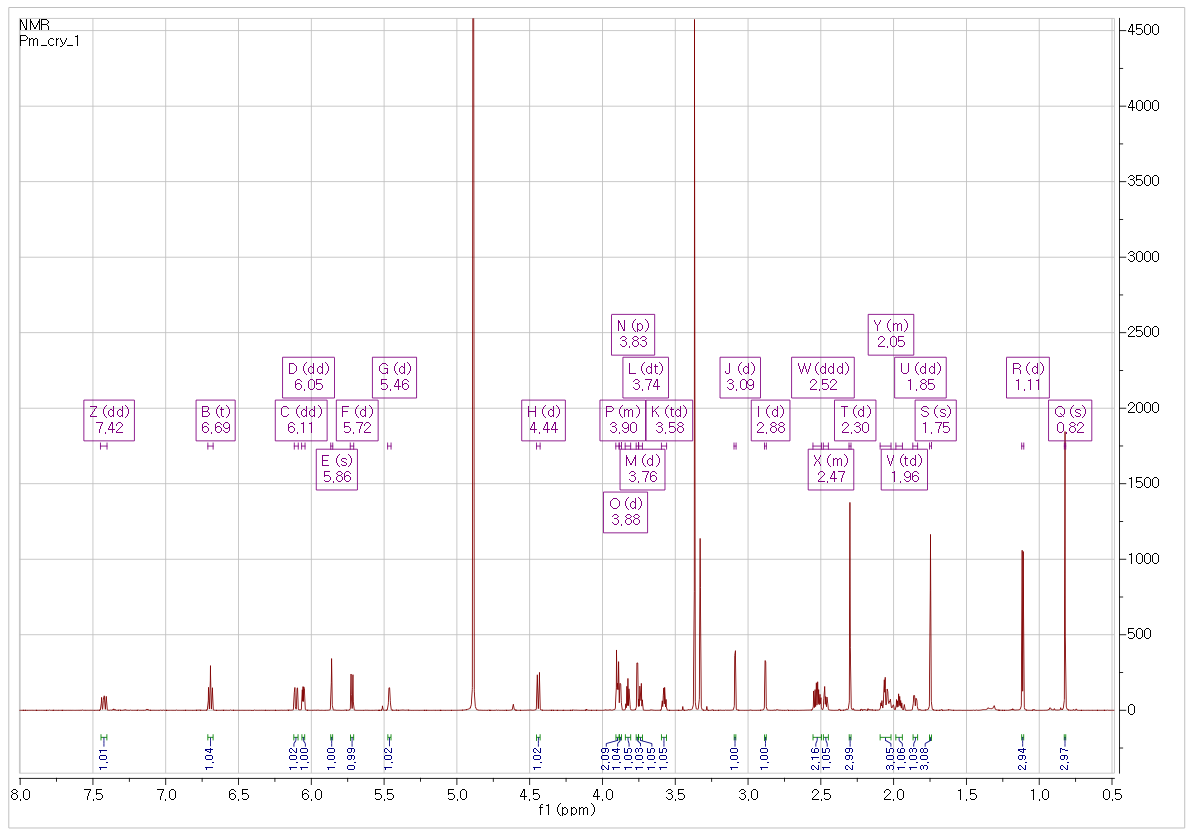
**

**Figure S8. The ^1^H NMR spectrum of roridin E (CD_3_OD, 850 MHz).**

**
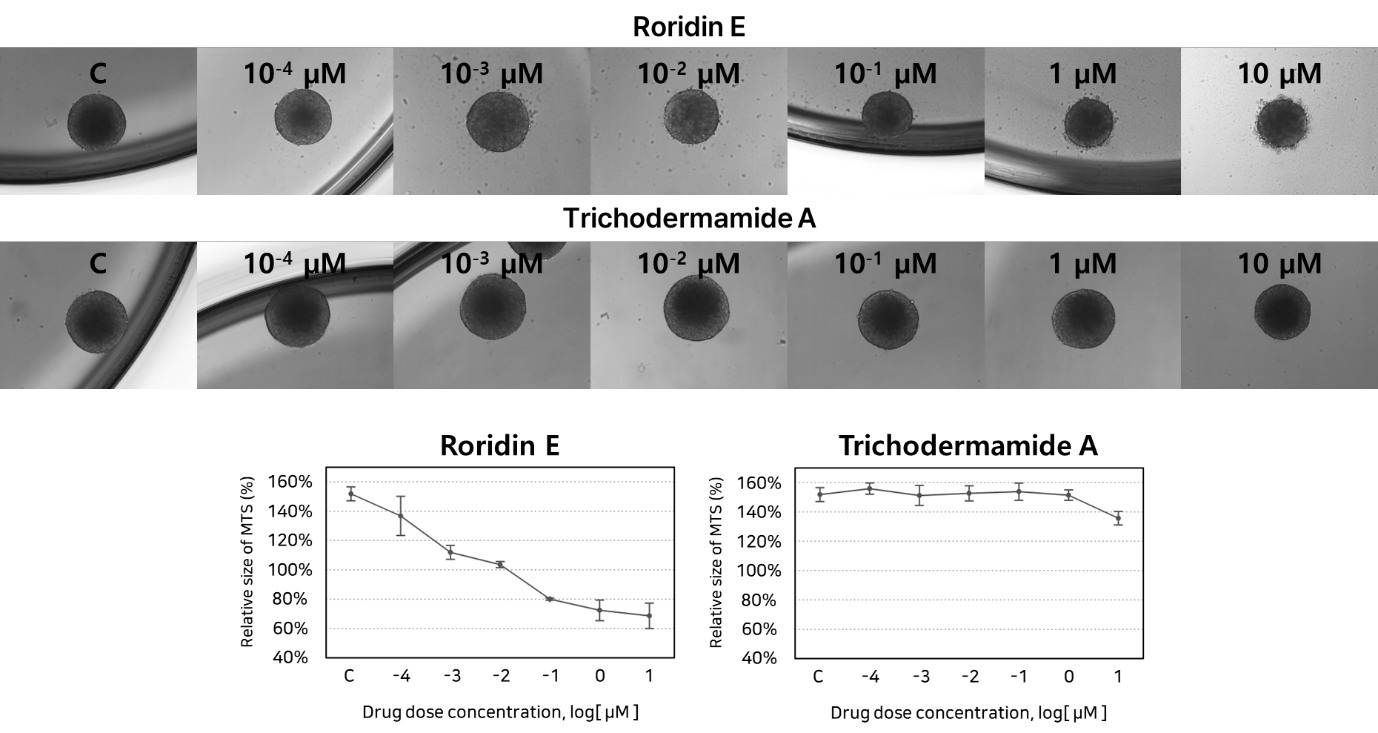
**

**Figure S9. Comparison of morphological changes in BT-474 MCTs treated with the active compound (roridin E) and the negative control (trichodermamide A), both of which were isolated from *Podostroma cornu-damae*.**

**
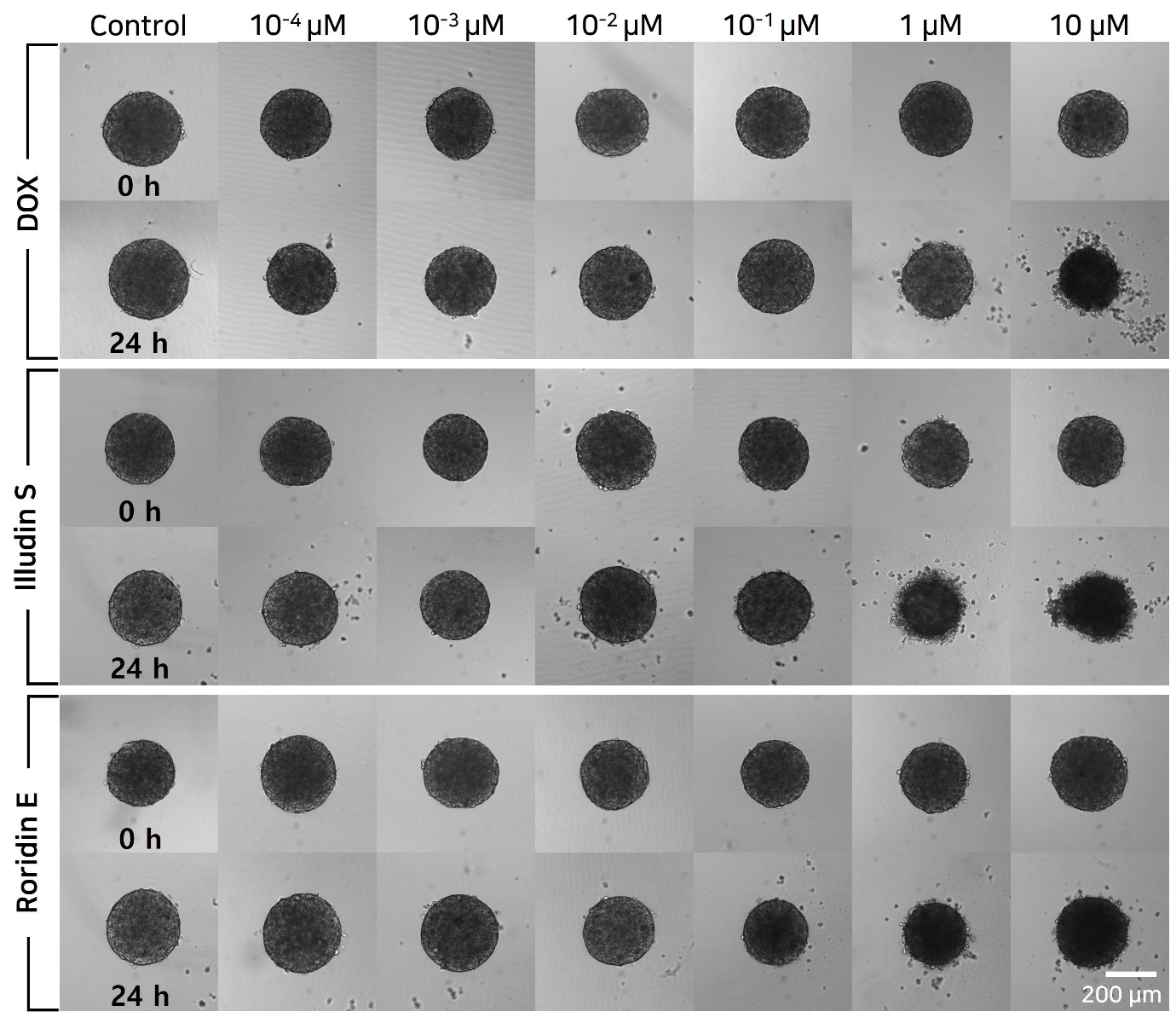
Figure S10. Morphological changes of BT-474 MCTs induced by drug response.** The BT-474 MCTs were treated with DOX, illudin S and roridin E respectively, ranging from 10^-4^ μM to 10 μM for 24 h.

**
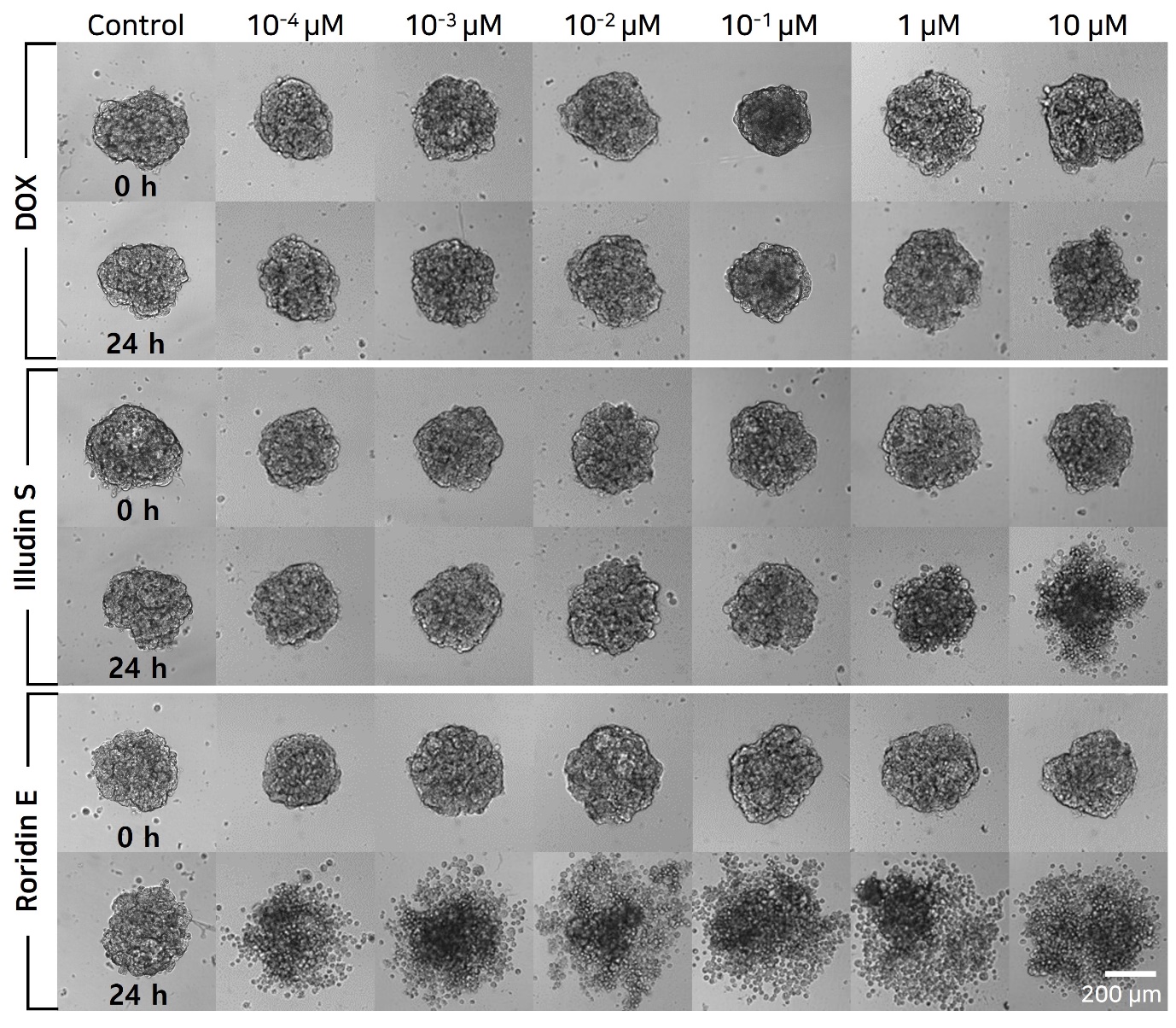
Figure S11. Morphological changes of MCF-7 MCTs induced by drug response.** The MCF-7 MCTs were treated with DOX, illudin S, and roridin E respectively, ranging from 10^-4^ μM to 10 μM for 24 h.

**
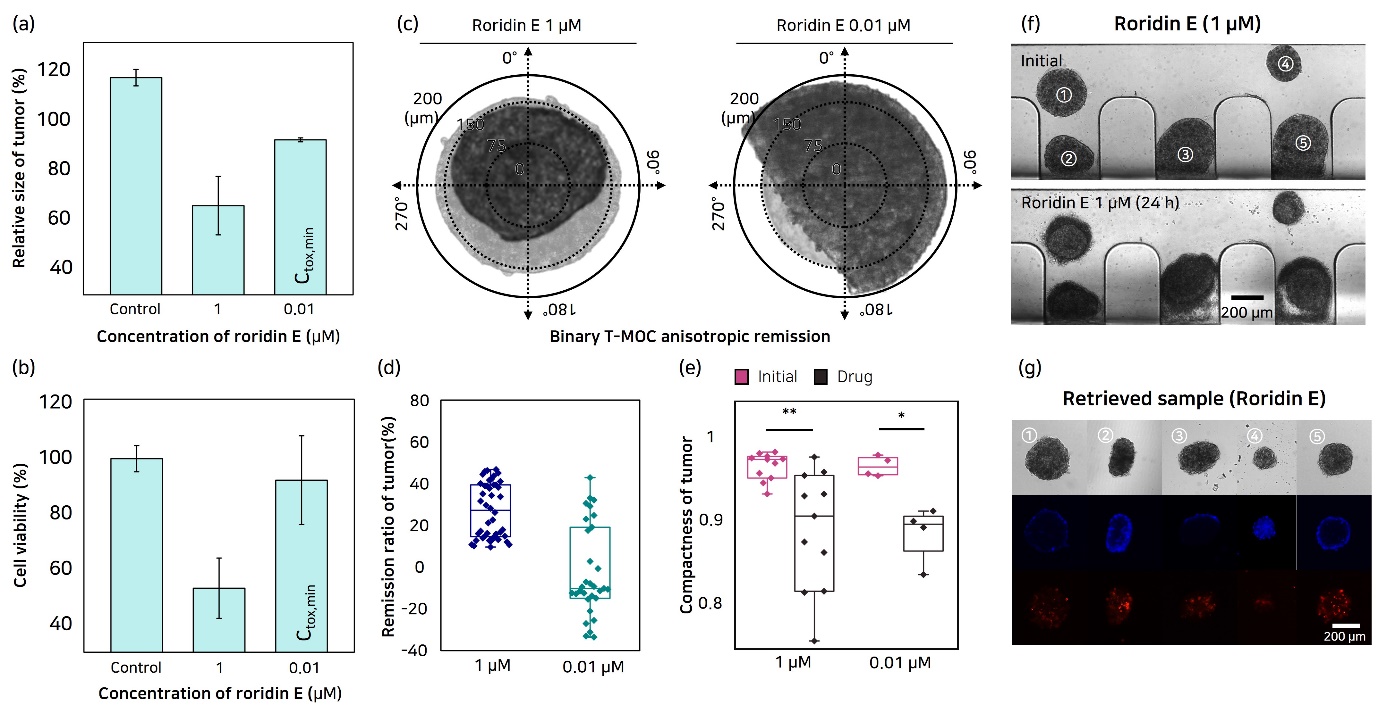
Figure S12. Roridin E drug test on binary T-MOC. a–b,** Relative size change and cell viability of BT-474 between initial and drug treated MCTs on binary T-MOC. **c**, Anisotropic remission in the binary T-MOC model. Geometric-based analysis of **d,** remission ratio (*n* = 6 (roridin E 1 μM), 5 (roridin E 0.01 μM)) and **e,** compactness (*n* = 8 (roridin E 1 μM), 5 (roridin E 0.01 μM)) of tumor in binary T-MOC model treated with chemotherapy by roridin E. **f,** Assessment of the anti-cancer drug efficacy of roridin E on binary T-MOC under 24 h physiological dynamics at concentrations of 1 μM. **g,** Live/Dead fluorescence staining image of the roridin E 1 μM treated retrieved sample from binary T-MOC (Nuclei (blue, hoechst 33342), dead cell (red, propidium iodide)) (mean ± SD, * *p* < 0.05, ** *p* < 0.01).


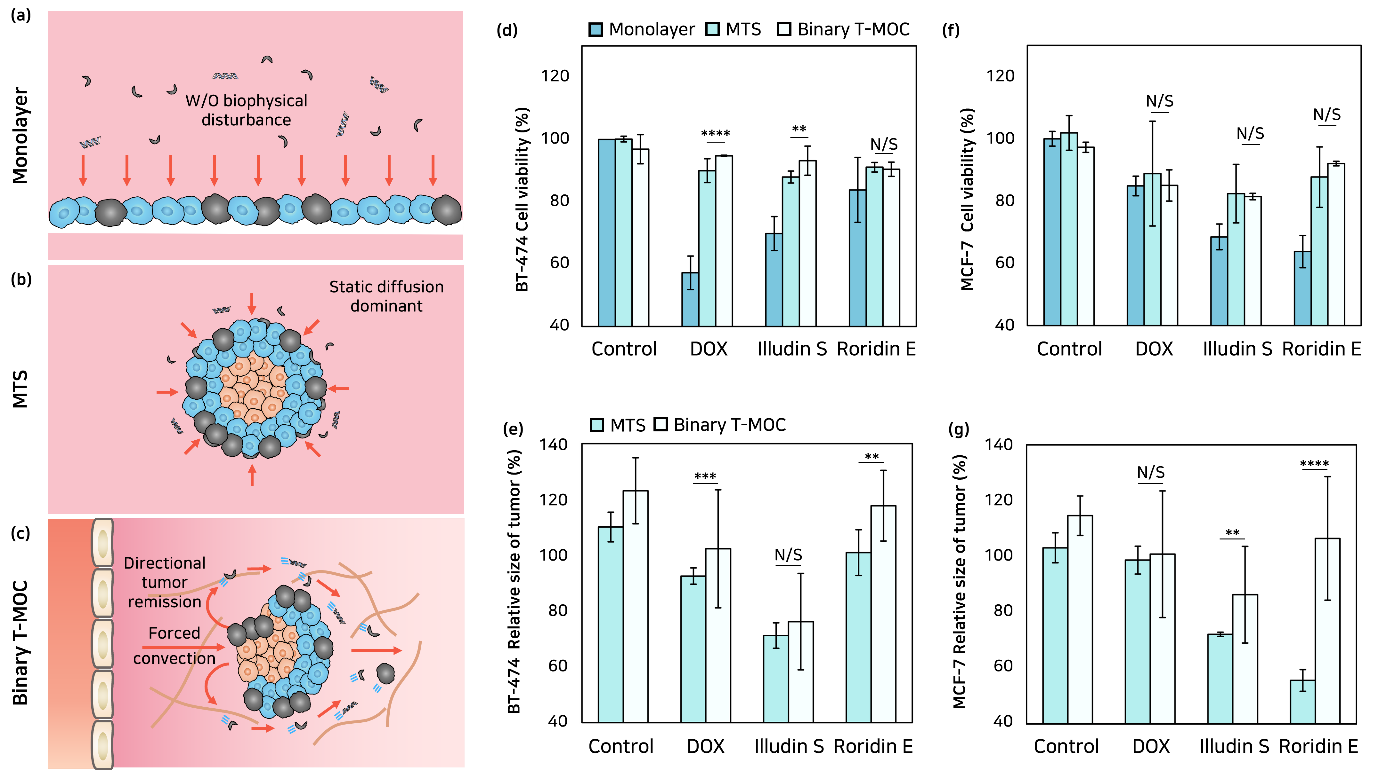
**Figure S13. Comparison of drug efficacy in in vitro NAMs models. a–c,** Schematic illustration of the cellular drug testing models, including the monolayer, MCTs, and binary T-MOC platforms. **d–g**, Drug efficacy analysis across models. Monolayers tended to overestimate cytotoxicity, while binary T-MOC showed similar or reduced efficacy compared to MCTs due to limited trans-endothelial delivery. Illudin S showed stronger remission than DOX, whereas roridin E caused severe structural disruption in MCF-7 but was less effective in BT-474 within the binary T-MOC. These findings underscore the importance of physiologically relevant models in anticancer drug evaluation. (BT-474 and MCF-7; mean ± SD, ***p* < 0.01, ****p* < 0.001, *****p* < 0.0001)


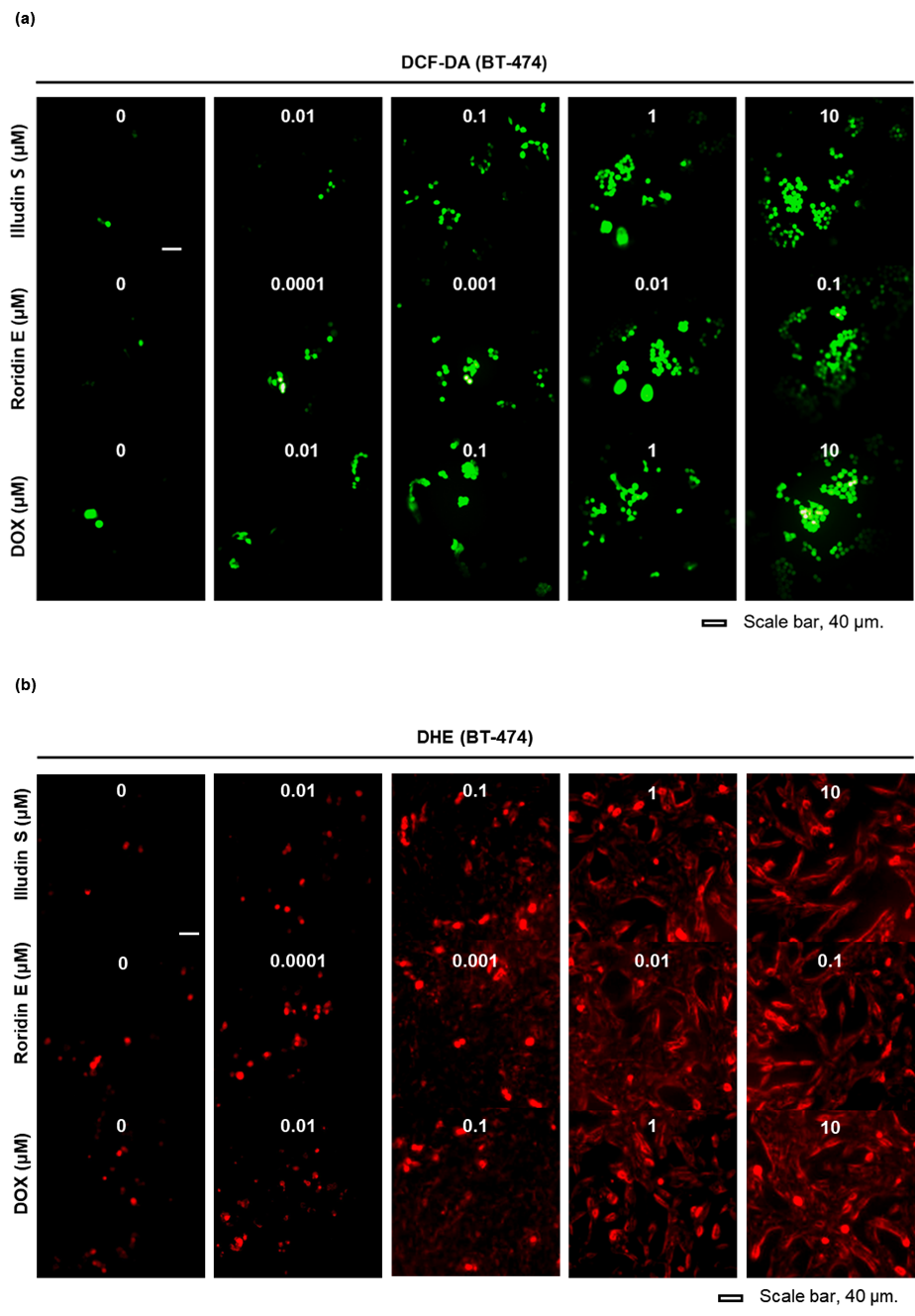


**Figure S14. Illudin S and roridin E induce oxidative stress in breast cancer cells BT-474.** Concentration-dependent production of reactive oxygen species (ROS) in BT-474 cells treated with illudin S, roridin E, and doxorubicin. **a,** Hydroxyl radicals were visualized by green fluorescence using H₂DCFDA staining. **b,** Superoxide anions were visualized by red fluorescence using dihydroethidium (DHE) staining. Compared to untreated controls, fluorescence intensity increased with drug concentration, indicating elevated oxidative stress.


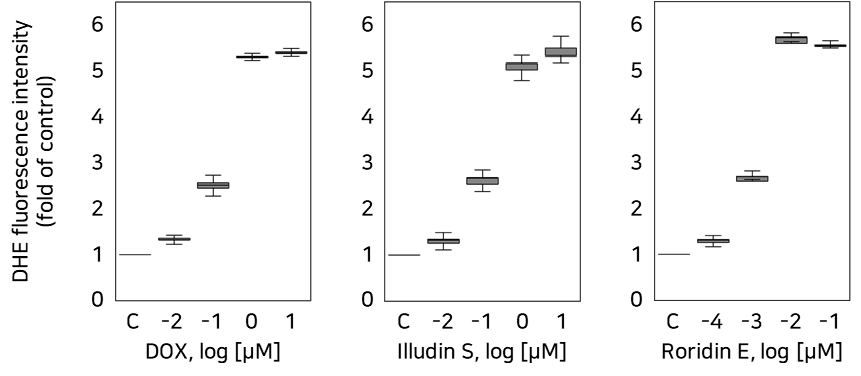


**Figure S15. Quantification of oxidative stress.** Intracellular reactive oxygen species (ROS) generation in BT-474 cells treated with DOX, illudin S, and roridin E for 24 h. ROS levels were quantified by red fluorescence using dihydroethidium (DHE) staining. ROS levels are shown as fold changes relative to control.

**
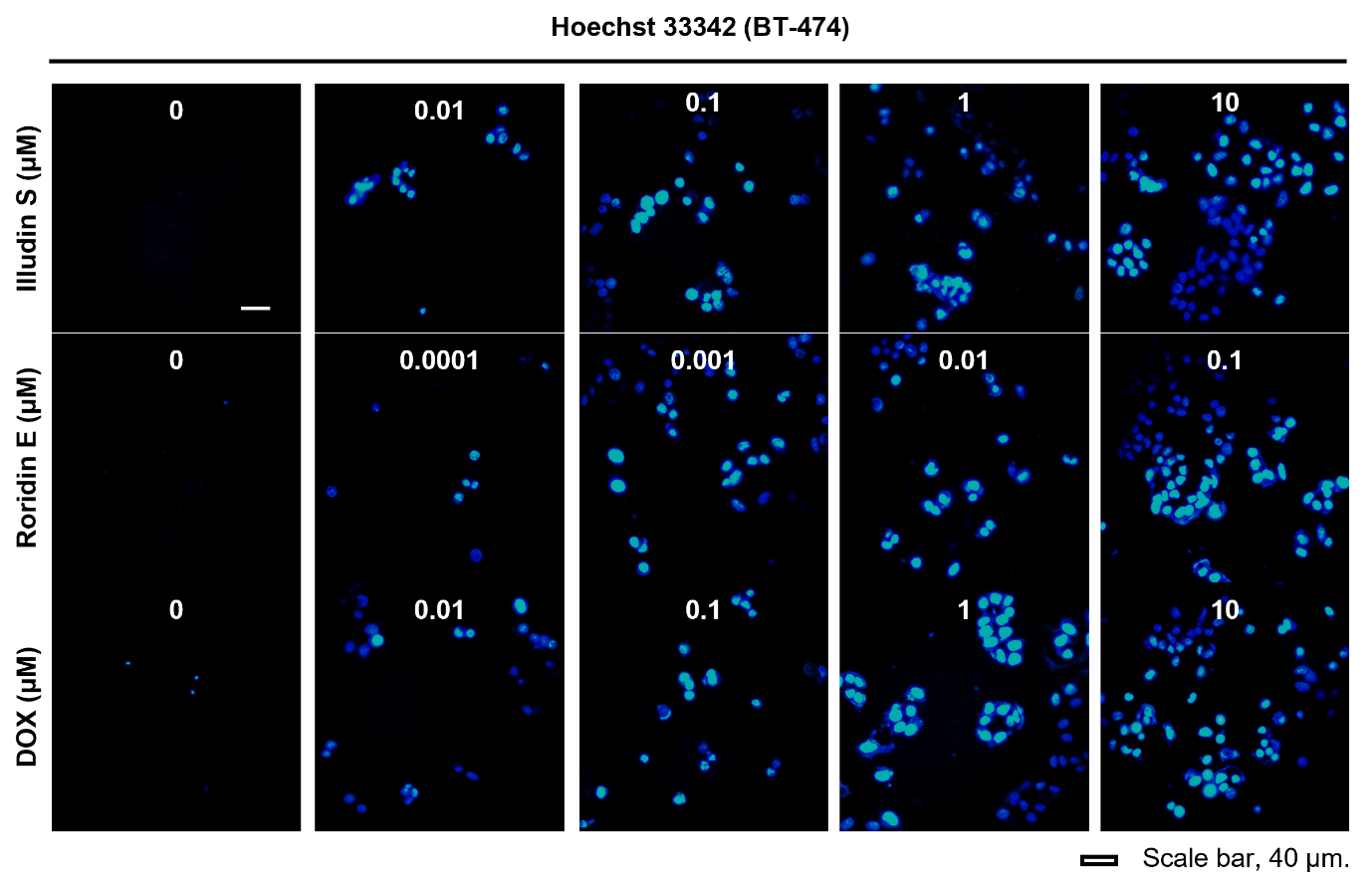
Figure S16. Illudin S and roridin E induce apoptotic nuclear changes in breast cancer cells BT-474.** Representative fluorescence images of BT-474 cells stained with Hoechst 33342 after treatment with illudin S, roridin E, or doxorubicin. Untreated control cells exhibited faint and uniform nuclear staining. In contrast, drug-treated cells showed bright, condensed, and fragmented nuclei, consistent with apoptotic nuclear morphology.
